# Supplementary material for: Cross-sectional study of the burden and determinants of non-medical and opportunity costs of accessing chronic disease care in rural Tanzania
Source: BMJ Open. 2024 Mar 29;14(3):e080466. doi: 10.1136/bmjopen-2023-080466 (PMC10982752; doi:10.1136/bmjopen-2023-080466)
Supplement: Supplementary data [file bmjopen-2023-080466supp001.pdf]

SUPPLEMENTARY MATERIALS

Supplementary tables

Table S1: Descriptive statistics, disaggregated according to whether patients were accompanied by a caregiver.

		Accompanier		No Accompanier		
Variable	Obs.	Mean	Std. Dev.	Mean	Std. Dev.	p-value
Sex (male)	383	**.264**	.441	**.39**	.488	**<0.001**
Education level	.	.	.	.	.	0.083
Never attended school	383	.073	.261	.042	.202	
Some primary school	383	.123	.329	.122	.327	
**Completed primary school**	383	**.475**	.5	**.545**	.498	
Some secondary school	383	.047	.212	.035	.184	
Completed secondary	383	.219	.414	.188	.391	
Completed college education	383	.047	.212	.049	.216	
Completed university education	383	.016	.124	.018	.134	
Working (last 12 months)	383	.159	.366	.298	.458	**<0.001**
Occupation	.	.	.	.	.	**<0.001**
Public servant	383	.039	.194	.056	.229	
Private formal sector	383	.018	.134	.097	.296	
**Subsistence farmer**	383	**.527**	.5	**.532**	.499	
Large-scale farming	383	.005	.072	.004	.06	
Self-employed/small business	383	.146	.354	.171	.377	
Self-employed/large business	383	.003	.051	.004	.06	
Taking care of home and/or children	383	.102	.303	.033	.179	
Student	383	.055	.228	.036	.186	
Retired	383	.065	.247	.041	.198	
Other	383	.039	.194	.027	.162	
Marital status	.	.	.	.	.	**0.004**
**Married**	383	**.627**	.484	**.655**	.476	
Living with partner	383	.042	.2	.021	.144	
Divorced	383	.008	.088	.007	.085	
Separated	383	.021	.143	.054	.227	
Widowed	383	.151	.359	.107	.309	
Never married	383	.151	.359	.155	.362	
Age	383	43.919	19.048	44.195	16.202	0.78
Health insurance	383	.368	.483	.402	.491	0.22
Any SHP	383	.428	.495	.484	.5	0.055
Any chronic condition	383	.337	.473	.297	.457	0.13
Type of chronic condition						
- Hypertension	129	.861	.348	.790	.408	0.078
- Diabetes	129	.217	.414	.244	.430	0.52
- Chronic kidney disease	129	.031	.174	.007	.086	0.040
- Epilepsy						
- Asthma	129	.0	.0	.015	0.121	0.16
- HIV	129	.039	.194	.025	.155	0.40
- TB	129	.031	.174	.047	.212	0.44
- Other	129	.0	.0	.007	.086	0.33

	129	.093	.292	.069	.254	0.37
Multiple chronic conditions	129	.24	.429	.198	.399	0.30
Prevented from working						**<0.001**
Completely prevented	129	.442	.499	.533	.5	
Never prevented	129	.186	.391	.067	.25	
Sometimes prevented	129	.372	.485	.4	.491	
Days missed work	129	7.69	11.565	4.341	8.708	**<0.001**
Facility level	.	.	.	.	.	**0.003**
Dispensary	383	.081	.273	.099	.299	
**Health centre**	383	**.742**	.438	**.651**	.477	
Hospital	383	.178	.383	.25	.433	
Closest facility visited	383	.791	.407	.888	.316	**<0.001**
Normal facility visited	383	.749	.434	.832	.374	**<0.001**
Transportation mode	.	.	.	.	.	**<0.001**
**Walk**	383	**.295**	.457	**.494**	.5	
Bicycle	383	.063	.243	.071	.257	
Your own motorbike	383	.078	.269	.033	.179	
Motorbike taxi	383	.36	.481	.28	.449	
Your own car	383	.06	.238	.018	.134	
Bus	383	.084	.277	.084	.278	
Bajaj	383	.037	.188	.015	.123	
A friend or family member brought me	383	.01	.102	.001	.038	
Other	383	.013	.114	.001	.038	
Travel time (return) (mins)	381	117.85	102.23	93.28	87.30	**<0.001**
Clinic time (mins)	378	134.45	103.06	129.94	104.50	0.46

Table S2 Non-medical costs of care-seeking and opportunity costs of illness, estimated using both the human capital method and self-reported lost earnings

	**Direct Non-Medical Cost**			**Indirect Non-Medical Cost**			**Total Non-Medical Cost**			**Opportunity costs of illness (CD patients)**		
	N	Mean (SD)	p-value	N	Mean (SD)	p-value	N	Mean (SD)	p-value	N	Mean (SD)	p-value
All	1675	1.39 (2.81)		1617	1.51 (2.13)		1550	2.93 (3.67)		498	21.88 (180.83)	
Chronic Condition			***			*			**			
No	1165	1.22 (2.68)		1117	1.48 (2.04)		1072	2.76 (3.50)			.	
Yes	510	1.76 (3.05)		500	1.58 (2.30)		478	3.32 (3.99)			.	
Multimorbidity			***						**			**
No	1568	1.35 (2.82)		1518	1.50 (2.14)		1454	2.89 (3.66)		399	22.70 (201.31)	
Yes	107	1.92 (2.54)		99	1.67 (1.91)		96	3.60 (3.77)		99	18.56 (35.14)	
Insurance status						***			***			*
Uninsured	1012	1.42 (3.08)		960	1.32 (1.72)		920	2.80 (3.72)		219	14.56 (24.80)	
Insured	662	1.34 (2.33)		656	1.79 (2.59)		629	3.12 (3.59)		279	27.62 (240.62)	
Sex						***						
Female	1074	1.43 (2.81)		1055	1.32 (1.80)		1017	2.76 (3.47)		341	11.87 (23.61)	
Male	601	1.31 (2.80)		562	1.86 (2.60)		533	3.26 (4.01)		157	43.61 (319.79)	
Accompanier			***						***			*
No	1320	1.18 (2.62)		1251	1.57 (2.29)		1211	2.79 (3.64)		376	23.87 (207.70)	
Yes	355	2.15 (3.32)		366	1.28 (1.40)		339	3.44 (3.72)		122	15.74 (23.61)	
Facility Level			***			***			***			
Dispensary	158	0.64 (1.50)		159	1.22 (2.29)		151	1.91 (2.99)		31	6.15 (17.39)	
Health Centre	1124	1.46 (3.01)		1122	1.45 (2.00)		1075	2.92 (3.66)		314	13.77 (26.53)	
Hospital	393	1.47 (2.58)		336	1.84 (2.40)		324	3.44 (3.89)		153	41.70 (323.77)	
Occupation						***			***			**
Formal	210	1.11 (2.29)		177	2.96 (3.38)		161	4.15 (4.22)		39	122.78 (639.09)	
Farmer	906	1.60 (3.24)		882	1.17 (1.24)		855	2.81 (3.59)		299	12.20 (25.25)	
Self-employed	288	1.20 (2.26)		280	1.83 (2.88)		272	3.09 (3.92)		58	10.92 (22.66)	
Other	266	1.10 (1.96)		273	1.33 (2.03)		257	2.44 (3.10)		97	16.87 (27.29)	

Table S3: Sensitivity analysis of Table 2 with new minimum hourly wage introduced from January 2023 (718 TZS ~ 0-30 USD)

	**Direct Non-Medical Cost**			**Indirect Non-Medical Cost**			**Total Non-Medical Cost**			**Estimated Lost Income (NCD patients)**		
	N	Mean (SD)	p-value	N	Mean (SD)	p-value	N	Mean (SD)	p-value	N	Mean (SD)	p-value
All	1675	1.39 (2.81)		1711	1.21 (0.78)		1638	2.61 (3.11)		534	14.62 (27.17)	
Chronic Condition			***			***			***			
No	1165	1.22 (2.68)		1189	1.12 (0.70)		1140	2.35 (2.89)			.	
Yes	510	1.76 (3.05)		522	1.41 (0.92)		498	3.20 (3.51)			.	
Multimorbidity			***			***			***			***
No	1568	1.35 (2.82)		1603	1.18 (0.74)		1534	2.54 (3.08)		423	13.34 (26.49)	
Yes	107	1.92 (2.54)		108	1.68 (1.17)		104	3.65 (3.37)		111	19.48 (29.22)	
Insurance status						***			***			**
Uninsured	1012	1.42 (3.08)		1029	1.10 (0.74)		984	2.53 (3.37)		238	18.11 (29.91)	
Insured	662	1.34 (2.33)		681	1.39 (0.82)		653	2.73 (2.69)		296	11.80 (24.44)	
Sex												
Female	1074	1.43 (2.81)		1092	1.22 (0.81)		1052	2.67 (3.15)		363	14.24 (26.85)	
Male	601	1.31 (2.80)		619	1.19 (0.73)		586	2.51 (3.05)		171	15.42 (27.90)	
Accompanier			***			***			***			**
No	1320	1.18 (2.62)		1335	1.18 (0.78)		1290	2.37 (2.92)		405	12.32 (24.71)	
Yes	355	2.15 (3.32)		376	1.33 (0.80)		348	3.50 (3.62)		129	21.82 (32.82)	
Facility Level			***			***			***			
Dispensary	158	0.64 (1.50)		166	0.73 (0.61)		158	1.36 (1.81)		31	5.31 (15.30)	
Health Centre	1124	1.46 (3.01)		1150	1.20 (0.73)		1101	2.68 (3.23)		327	15.80 (28.14)	
Hospital	393	1.47 (2.58)		395	1.43 (0.90)		379	2.92 (3.07)		176	14.05 (26.73)	
Occupation						**						***
Formal	210	1.11 (2.29)		227	1.14 (0.69)		207	2.25 (2.61)		51	2.78 (7.28)	
Farmer	906	1.60 (3.24)		913	1.20 (0.81)		884	2.81 (3.53)		314	14.67 (26.68)	
Self-employed	288	1.20 (2.26)		288	1.22 (0.81)		280	2.45 (2.67)		64	11.49 (23.91)	
Other	266	1.10 (1.96)		278	1.31 (0.74)		262	2.40 (2.32)		100	20.58 (32.65)	

Table S4: Sensitivity analysis of mixed-effects linear regression for total non-medical cost, including a random intercept for administrative district

	Total Non-Medical Cost	95% CI
Health Insurance	0.136**	0.05 – 0.23
	(0.046)	
Hypertension	0.133*	0.005 – 0.26
	(0.065)	
Multiple Chronic Conditions	0.32***	0.14 – 0.50
	(0.091)	
Sex (= male)	0.01	(0.08) – 0.10
	(0.044)	
Median Age	-0.077	(0.17) – 0.02
	(0.049)	
Higher education	-0.115**	(0.21) – (0.018)
	(0.049)	
Employed (last 12 months)	0.027	(0.07) – 0.13
	(0.0561)	
Closest facility	-0.898***	(1.00) – (0.79)
	(0.0584)	
Accompanier	0.390***	0.25 – 0.45
	(0.051)	
Hospital	0.649***	0.49 – 0.81
	(0.083)	
Health Center	0.546***	0.40 – 0.69
	(0.074)	
Hospital#Hypertension	0.321**	0.08 – 0.56
	(0.123)	
Constant	8.312***	8.13 – 8.49

	(0.09)
Observations	1,637
Clusters (districts)	2

Standard errors in parentheses
95% CI: negative numbers in parentheses
Note: * p <.05 ** p <.01 *** p <.001

Author reflexivity statement

1. How does this study address local research and policy priorities?

This study directly addresses the as-yet underappreciated issue of indirect costs of accessing healthcare in Tanzania, which disproportionately affects Tanzania's majority rural population. This study brings attention to this local issue, and makes recommendations for improving the equity of access to NCD care in Tanzania.

2. How were local researchers involved in study design?

Local researchers KT and GM were involved in the design of the greater research project within which this study is nested, in addition to having been involved in the design of the methodology for the present study. They both have rich experience in international research partnerships, completing and having completed their doctorate at the University of Basel, respectively. They represent a gender- and seniority-balanced local research team composition.

3. How has funding been used to support the local research team?

This study was nested within the broader "Health systems governance for an inclusive and sustainable social health protection in Ghana and Tanzania" study, funded by the Swiss programmes for research on global issues for development that was jointly obtained by Switzerland- and Tanzania-based co-PIs, Prof. Dr. Fabrizio Tediosi and Dr. Sally Mtenga respectively. Throughout the 6-year lifespan of this project, this grant supported equal numbers of Switzerland- and Tanzania-based doctoral students and researchers.

4. How are research staff who conducted data collection acknowledged?

This study recruited research assistants to perform data collection, who are appropriate credited in the acknowledgements section.

5. Do all members of the research partnership have access to study data?

All members of the partnership have access to the study data.

6. How was data used to develop analytical skills within the partnership?

Both Switzerland- and Tanzania-based collaborated on the design of data collection tools, further developing their skills in programming ODK forms. Both Tanzania- and Switzerland-based co-authors had the opportunity to follow courses at the University of Basel to develop the analytical and data analysis skills, while directly working with the data stemming from this study.

7. How have research partners collaborated in interpreting study data?

Authors BH and KT worked together on the cleaning and initial interpretation of study data.

8. How were research partners supported to develop writing skills?

The first authors of this manuscript are junior academics, who received support from senior academic FT throughout the writing process.

9. How will research products be shared to address local needs?

This work has been directly disseminate via two stakeholder panel discussions and presentations with stakeholders and policymakers from the Ministry of Health, Community Development, Elderly and Children, the President's Office for Regional Administration and Local Government, and the Tanzanian National Health Insurance Fund. Both events were attended by local journalists and media, which helped ensure a wider local dissemination of findings.

10. How is the leadership, contribution and ownership of this work by LMIC researchers recognised within the authorship?

Authors RM and NO worked as part of the senior authorship team in developing this manuscript, and their contribution has been recognised as joint first and joint last authors respectively. We have specifically included researchers based in the global south (RM and NO) within the senior authorship team as joint first and joint last authors. We acknowledge, however, that the authorship team is predominantly based in high-income countries. The primary reason for this is that the initiative has been driven from the perspective of journal editors with insight on how to develop transparent and implementable guidelines to assess manuscript submissions from international partnerships.

11. **How have early career researchers across the partnership been included within the authorship team?**

We have included early career researchers (CK, SS & MS) within the authorship team. They attended all the workshops, contributed to the literature review and evidence synthesis and to development of the consensus recommendations. We acknowledge that they are based in high-income countries.

12. How has gender balance been addressed within the authorship?

Three co-authors are men (BH, KT and FT) and two co-authors women (AV, GM).

13. How has the project contributed to training of LMIC researchers?

All researchers based in Tanzania (KT and GM) received or are receiving doctoral training and education in Switzerland, at the University of Basel, financed by the Research for development grant described in point number three.

14. How has the project contributed to improvements in local infrastructure?

This project did not directly contribute to improving local infrastructure.

15. What safeguarding procedures were used to protect local study participants and researchers?

All participants provided informed consent prior to participation. Research protocols were critically reviewed and approved by the institutional review board of the Ifakara Health Institute and the National Institute for Medical Research of Tanzania.
