# Supplementary material for: Cross-sectional study of the burden and determinants of non-medical and opportunity costs of accessing chronic disease care in rural Tanzania
Source: BMJ Open. 2024 Mar 29;14(3):e080466. doi: 10.1136/bmjopen-2023-080466 (PMC10982752; doi:10.1136/bmjopen-2023-080466)
Supplement: Author's manuscript [file bmjopen-2023-080466.draft_revisions.pdf]

BMJ Open is committed to open peer review. As part of this commitment we make the peer review history of every article we publish publicly available.

When an article is published we post the peer reviewers' comments and the authors' responses online. We also post the versions of the paper that were used during peer review. These are the versions that the peer review comments apply to.

The versions of the paper that follow are the versions that were submitted during the peer review process. They are not the versions of record or the final published versions. They should not be cited or distributed as the published version of this manuscript.

BMJ Open is an open access journal and the full, final, typeset and author-corrected version of record of the manuscript is available on our site with no access controls, subscription charges or pay-per-view fees (http://bmjopen.bmj.com).

If you have any questions on BMJ Open's open peer review process please email info.bmjopen@bmj.com

BMJ Open

A cross-sectional study of the burden and determinants of indirect costs of accessing NCD care in rural Tanzania

Journal:	*BMJ Open*
Manuscript ID	bmjopen-2023-080466
Article Type:	Original research
Date Submitted by the Author:	02-Oct-2023

Complete List of Authors:	Verjans, Anna; Swiss Tropical and Public Health Institute; University of Basel<br>Hooley, Brady; Swiss Tropical and Public Health Institute; University of Basel, Faculty of Medicine<br>Tani, Kassimu; Swiss Tropical and Public Health Institute; University of Basel<br>Mhalu, Grace; Ifakara Health Institute<br>Tediosi, Fabrizio; Swiss Tropical and Public Health Institute; University of Basel

Keywords:	Health policy < HEALTH SERVICES ADMINISTRATION & MANAGEMENT, Chronic Disease, HEALTH ECONOMICS, Health Services Accessibility

SCHOLARONE™
Manuscripts

I, the Submitting Author has the right to grant and does grant on behalf of all authors of the Work (as defined in the below author licence), an exclusive licence and/or a non-exclusive licence for contributions from authors who are: i) UK Crown employees; ii) where BMJ has agreed a CC-BY licence shall apply, and/or iii) in accordance with the terms applicable for US Federal Government officers or employees acting as part of their official duties; on a worldwide, perpetual, irrevocable, royalty-free basis to BMJ Publishing Group Ltd ("BMJ") its licensees and where the relevant Journal is co-owned by BMJ to the co-owners of the Journal, to publish the Work in this journal and any other BMJ products and to exploit all rights, as set out in our licence.

The Submitting Author accepts and understands that any supply made under these terms is made by BMJ to the Submitting Author unless you are acting as an employee on behalf of your employer or a postgraduate student of an affiliated institution which is paying any applicable article publishing charge ("APC") for Open Access articles. Where the Submitting Author wishes to make the Work available on an Open Access basis (and intends to pay the relevant APC), the terms of reuse of such Open Access shall be governed by a Creative Commons licence – details of these licences and which Creative Commons licence will apply to this Work are set out in our licence referred to above.

Other than as permitted in any relevant BMJ Author's Self Archiving Policies, I confirm this Work has not been accepted for publication elsewhere, is not being considered for publication elsewhere and does not duplicate material already published. I confirm all authors consent to publication of this Work and authorise the granting of this licence.

For peer review only

A cross-sectional study of the burden and determinants of indirect costs of accessing NCD care in rural Tanzania

For peer review only

For peer review only - http://bmjopen.bmj.com/site/about/guidelines.xhtml

Key words: Noncommunicable diseases, Outpatient care, Tanzania, Health service provision, Indirect health expenditure, Self-referral

ABSTRACT (300 words)

Objectives: Countries in sub-Saharan Africa are seeking to improve access to healthcare through health insurance schemes. However, insured patients continue to bear indirect costs such as transportation and lost workdays. The burden of indirect costs is particularly high for people with non-communicable diseases (NCDs) who require regular healthcare. This study quantified the indirect costs faced by NCD patients compared to other patients in Tanzania, identified factors that drive these differences, and explored the relationship between engagement in care and NCD-related work limitations.

Methods: From November 2020 to January 2021, we conducted a cross-sectional patient survey at 35 healthcare facilities in rural Tanzania. Using the human capital approach to value the indirect costs of seeking healthcare, we employed multilevel linear regression to analyze the impact of chronic diseases and health insurance on indirect costs and negative binomial regression to investigate the association between retention in care and NCD-related work limitations.

Results: Among 1748 patients surveyed, 534 had at least one NCD, 20% of which had comorbidities. The average indirect cost of accessing care was \$2.21 (\$0.059 - \$27.14) per patient. NCD patients incurred significantly higher indirect costs, with an average monthly illness-related opportunity cost of \$10.19 (\$0 - \$59.34). Factors associated with higher indirect costs included multimorbidities, hypertension, health insurance, and seeking care at hospitals rather than lower-level facilities. Patients seeking hypertension care at hospitals experienced 35% higher costs compared to those visiting other facilities. Additionally, patients with comorbidities, older age, lower education levels, and those requiring medication more frequently lost workdays.

Conclusion: Outpatient care in Tanzania entails considerable indirect costs, particularly for individuals with NCDs. Despite having health insurance, NCD patients attending hospital outpatient care face higher financial burdens compared to other patients. To alleviate these costs, we recommend improving the availability and quality of NCD care at lower-level facilities.

For peer review only - http://bmjopen.bmj.com/site/about/guidelines.xhtml

KEY MESSAGES

- What is already known?
    - Tanzania has been trying to expand health insurance coverage as a means of reducing the burden of out-of-pocket health expenditure on its population. However, for its largely rural population, the time and cost of travelling to seek care can represent a substantial portion of the total costs of seeking healthcare and are unaffected by health insurance.
- What does this study add?
    - This study specifically investigates the impact of health insurance and non-communicable diseases upon the non-medical costs incurred by patients when seeking healthcare.
- How may this study affect research, practice or policy?
    - We found that patients with health insurance and/or non-communicable diseases incur significantly higher non-medical costs when seeking care relative to other patients. Inequitable provision of NCD services to rural communities is likely the main driver of these costs and could be mitigated by strengthening the capacity of primary care services to provide NCD care.

For peer review only

For peer review only - http://bmjopen.bmj.com/site/about/guidelines.xhtml

INTRODUCTION

Universal health coverage (UHC) aims to ensure "that people have access to the healthcare they need without suffering financial hardship".[1] Country-level policies have so far focused on reducing the direct medical out-of-pocket cost of healthcare services and drugs, without addressing the non-medical costs of illness such as loss of income and the cost of travelling to access healthcare.[2] Utilizing healthcare induces costs that extend beyond the medical costs covered by health insurance and involves transportation and income losses for both patients and any accompanying caregivers.[2]

This narrow focus of social health protection and service provision is particularly problematic for health systems in low- and middle-income countries (LMICs), whose design prioritizes the management of communicable diseases where the household economic burden associated with illness is more readily characterised by discrete, yet still potentially catastrophic disease events.[3, 4] On the other hand, despite mounting evidence of the increased burden of NCDs in LMICs, care for NCDs remains centralized at hospitals and many basic medicines for the treatment of hypertension and diabetes are not readily available at lower-level health facilities.[5] As a result, the financial burden of seeking NCD care tends to be greater than that of seeking acute or communicable disease care, in that NCD patients may need to utilise healthcare services more frequently and seek centralised care at hospitals in order to ensure that they receive the care they require.[6, 7]

The issue of burdensome non-medical healthcare costs has been studied in sub-Saharan Africa (SSA), particularly in the context of HIV and tuberculosis care.[8-10] As with NCDs, the chronic nature of HIV and TB management requires patients to visit healthcare facilities and renew prescriptions on a 1-3 month basis, meaning that patients must regularly pay for transport to the clinic and spend time accessing care that could have otherwise been spent working.[9, 10] The accumulation of travel costs and productivity loss associated with illness and care seeking can therefore surpass the direct cost of care, representing 55% of costs for HIV infections and 71% of costs for HIV/TB coinfections.[10] However, the introduction of a decentralised community-based HIV care program in Tanzania substantially reduced both the indirect and direct costs of accessing HIV care, indicating that such an intervention or policy implementation could yield promising results for reducing the indirect costs of care seeking for people living with chronic conditions.[11]

While research on the indirect cost of seeking NCD care in SSA is more limited, past work has indicated that some patients incur substantial indirect costs, or even completely forgo seeking care in order to avoid the impoverishing effect of direct medical or non-medical costs of care.[6, 12-15] In Mali,

For peer review only

the indirect costs of diabetes mellitus were estimated to make up 61% of the total costs,[6] while in rural Malawi the direct costs of NCDs formed the largest cost element, with 56.8% of the total.[16] While not directly comparable, their findings demonstrate a substantial burden of indirect costs on patients.

These indirect costs form a barrier that can therefore affect uptake and adherence to treatment, which for chronic conditions can contribute to severe long-term effects. In this regard, prevention and early treatment of NCDs can avoid the need for more expensive treatment procedures in the future, caused by more severe illness, which usually also requires more frequent follow-up. For instance, a study found higher transportation costs and longer waiting times for ART compared to pre-ART patients in rural South Africa.[8] Besides early onset of treatment, absenteeism and short-term disability among chronic disease patients can also be reduced by improving medication adherence.[17] This highlights the potential economic gain of implementing alternative care models that lower the financial burden for NCD patients by avoiding future opportunity costs.

The human capital method and friction cost method are two of the most commonly used methods for measuring opportunity costs. The former measures opportunity costs in terms of the value of lost income, including future productivity losses, while the latter only considers these losses up until the market finds a replacement.[18] The human capital method takes a patient perspective and is often preferred over the friction cost method since it is better able to account for presenteeism, although it is sometimes criticized for overestimating the indirect costs.[19] To compensate for lost working time and income, households often adopt various 'distress financing' strategies, such as borrowing money or selling assets, especially in informal economies.[20] However, the dependence of agricultural economies on seasonality means that sick days do not necessarily translate into lost working days.[20] Income losses in the agricultural sector are sometimes approximated by the minimum daily wage of the country or by taking the GNI per capita and transforming it into a daily value,[6, 15] the latter of which may inflate indirect costs.[21]

Previous work in Tanzania showed that people with cardiovascular risk factors incur substantial non-medical and opportunity costs, yet there is little evidence regarding the actual non-medical costs of seeking NCD care in Tanzania.[22] Considering the potential burden of non-medical and opportunity costs of seeking healthcare, this study surveyed outpatients from 35 health facilities in two rural districts in order to estimate these costs in the rural Tanzanian context and specifically compared NCD to non-NCD patients.

For peer review only - http://bmjopen.bmj.com/site/about/guidelines.xhtml

METHODS

This study is part of the Chronic Conditions Household and Exit Survey in Tanzania (CHEST), a cross-sectional outpatient and household survey in the rural Same and Kilombero districts conducted between November 2020 and January 2021. This survey recruited 784 household members and 1748 outpatients above the age of 18, however this study used only outpatient data on the reason for their health facility visit, time and cost of transport to the facility, time spent at the clinic and what they expect they would have earned if they had not sought care that day. The sampling strategy and full details of the CHEST survey have been described in previous work.[23]

The outcome of interest was the total non-medical cost of seeking healthcare services, comprising both direct non-medical costs and indirect costs. In this case, direct non-medical cost included transportation fare while the indirect costs included the opportunity cost associated with travel and clinic time. We estimated indirect cost using the human capital method, utilizing the national minimum hourly wage for the agricultural sector (512.82 TZS ~ 0.22 USD), the largest occupational group in our sample.[24] However, some salaried participants reported lost income, so we performed a sub-analysis of this data, while imputing human capital-based opportunity costs for those participants who did not provide estimates for their forgone wages (see supplementary materials). All costs were converted to 2020 US Dollars (1 USD = 2277 TZS).[25] For survey participants who claimed to have a chronic condition, we additionally estimated the cost of lost work over the past month due to illness, based on the number of days they reported being unable to work.

We used a mixed-effects multivariate linear regression to investigate the association between non-medical costs and insurance status and NCD diagnoses, while controlling for various demographic factors such as gender, age, education, occupation, accompaniment by a caregiver, level of healthcare facility, and residential proximity to the facility. We included a random intercept term to account for between-district variability, and log transformed non-medical costs in order to maintain the assumptions of linear regression. Additionally, we included an interaction term to examine the impact of hospital care on patients with hypertension.

On January 1st, 2023, the Government of Tanzania introduced a new wage order, increasing the hourly wage of agriculture workers by approximately 40%, from 512.82 TZS to 718 TZS.[24, 26] We therefore performed a sensitivity analysis to investigate the impact of higher wages on indirect health expenditure by using the new minimum wage in human capital method estimates of opportunity costs.

For peer review only

To evaluate the impact of reduced work capacity on NCD patients, we used negative binomial regression[1] to determine the association between engagement in care and the number of days missed from work over the last 30 days. The predictor variables of interest included the prescription of medication during the last visit, medication adherence over the last week, and the presence of multiple chronic conditions, with controls for age, gender, education, and occupation. We used both STATA and R for analysis.[27, 28]

Ethical clearance

This studied obtained ethical clearance from the Ifakara Health Institute (IHI) Institutional Review Board (IHI/IRB/EXT/No:35–2020) and the Tanzanian National Institute for Medical Research (NIMR/HQ/R.8a/Vol.IX/3518).

Patient and public involvement statement

We first involved the public by engaging with healthcare providers prior to the design of this study, where informal conversations with providers at rural hospitals and health centres indicated that few NCD services are available at lower-level health facilities and that patients with NCDs must frequently be referred or self-refer over substantial distances for relatively basic NCD services and prescription medicines. These providers lamented that the need to seek NCD care in hospitals forms both a substantial barrier to care that prevents would-be patients from being retained in care in a timely manner, and that those who are retained in care face substantial non-medical costs.

The findings of this work were directly presented to Tanzanian stakeholders and policymakers via workshops and presentations in Dodoma and Dar es Salaam, where we engaged with attendees from the Ministry of Health, National Health Insurance Fund, Improved Community Health Fund and President's Office for Regional Administration and Local Government.

RESULTS

We recruited 1748 outpatients, of which 63.73% were women, 64.87% married, 52.97% completed primary school, 53.09% subsistence farmers and a mean age of 44 years (Table 1). Forty percent were health insurance beneficiaries and about 30% had a chronic condition. Hypertension was the most

[1] Poisson regression models were tested against negative binomial models using likelihood ratio tests. Given the significant overdispersion, the negative binomial model was preferred, which is in line with analysis standards for absenteeism data.

For peer review only - http://bmjopen.bmj.com/site/about/guidelines.xhtml

commonly reported NCD (81%) and more than 20% of NCD patients had comorbid NCDs. NCD patients lost an average of five working days due to their illness during the month before the survey, with almost 10% completely unable to work.

Table 1: Descriptive statistics of total sample

Variable	N	Mean	SD
Sex (male)	1748	36.3%	48.1%
Education level	.	.	.
Never attended school	1748	4.9%	21.6%
Some primary school	1748	12.2%	32.7%
Completed primary school	1748	53%	49.9%
Some secondary school	1748	3.8%	19.1%
Completed secondary	1748	19.5%	39.6%
Completed college education	1748	4.9%	21.5%
Completed university education	1748	1.8%	13.2%
Working (last 12 months)	1748	26.8%	44.3%
Occupation	.	.	.
Public servant	1748	5.2%	22.2%
Private formal sector	1748	8%	27.1%
Subsistence farmer	1748	53.1%	49.9%
Large-scale farming	1748	0.4%	6.3%
Self-employed/small business	1748	16.6%	37.2%
Self-employed/large business	1748	0.3%	5.9%
Taking care of home and/or children	1748	4.8%	21.4%
Student	1748	4%	19.6%
Retired	1748	4.6%	21%
Other	1748	3%	17%
Marital status	.	.	.
Married	1748	64.9%	47.8%
Living with partner	1748	2.6%	15.8%
Divorced	1748	0.7%	8.6%
Separated	1748	4.7%	21.2%
Widowed	1748	11.7%	32.1%
Never married	1748	15.4%	36.1%
Age	1748	44.134	16.861
Health insurance	1747	39.5%	48.9%
Any SHP	1748	47.1%	49.9%
Any chronic condition	1748	30.5%	46.1%
Type of chronic condition			
- Hypertension	534	80.7%	39.5%
- Diabetes	534	23.8%	42.6%
- Chronic kidney disease	534	1.5%	11.4
- Epilepsy	534	1.3%	10.6%
- Asthma	534	2.8%	16.5%
- HIV	534	4.3%	20.3%
- TB	534	0.06%	7.5%
- Other	534	7.5%	26.3%
Multiple chronic conditions	534	20.8%	40.6%
Prevented from working			

For peer review only

For peer review only - http://bmjopen.bmj.com/site/about/guidelines.xhtml

Completely prevented	534	9.6%	29.4%
Never prevented	534	51.1%	50%
Sometimes prevented	534	39.3%	48.9%
Days missed work (last month)	534	5.15	9.574
Facility level	.	.	.
Dispensary	1748	9.5%	29.3%
Health centre	1748	67.1%	47%
Hospital	1748	23.4%	42.3%
Closest facility visited	1748	81.4%	38.9%
Normal facility visited	1748	86.7%	34%
Accompanied to the facility	1748	21.9%	41.4%
Transportation mode	.	.	.
Walk	1746	45.1%	49.8%
Bicycle	1746	6.9%	25.4%
Your own motorbike	1746	4.3%	20.3%
Motorbike taxi	1746	29.8%	45.7%
Your own car	1746	2.7%	16.4%
Bus	1746	8.4%	27.8%
Bajaj	1746	2%	14%
A friend or family member brought me	1746	0.3%	5.9%
Other	1746	0.4%	6.3%
Travel time (return) (mins)	1744	98.64	91.31
Clinic time (mins)	1714	130.94	104.17

Most NCD patients appeared adherent to their treatment and follow-up schedule, with 77% seeking care within the last month and 89% having prescription medicines of which 76% took their medicines within the last seven days. Most participants were recruited from a health centre (67.11%) and 21.91% were accompanied to the facility, mostly by one of their children or their partner. Most participants travelled by foot (45%) or motorbike taxi (30%) and for more than an hour and a half of return trip travel. In addition to travel time, patients spent more than two hours at the facility, including both waiting and consultation time (Table 2). The average travel time was longer when attending health centres or hospitals compared to dispensaries.

For peer review only

For peer review only - http://bmjopen.bmj.com/site/about/guidelines.xhtml

Table 2: Direct non-medical and indirect non-medical costs of care-seeking, estimated using the human capital method.

	Direct Non-Medical Cost			Indirect Non-Medical Cost			Total Non-Medical Cost			Estimated Lost Income (NCD patients)		
	N	*Mean (SD)*	*p-value*	*N*	*Mean (SD)*	*p-value*	*N*	*Mean (SD)*	*p-value*	*N*	*Mean (SD)*	*p-value*
All	1675	1.39 (2.81)		1711	0.87 (0.56)		1638	2.26 (3.02)		534	10.44 (19.41)	
Chronic Condition			***			***			***			
- No	1165	1.22 (2.68)		1189	0.80 (0.50)		1140	2.03 (2.82)			.	
- Yes	510	1.76 (3.05)		522	1.01 (0.66)		498	2.79 (3.36)			.	
Multimorbidity			***			***			***			***
- No	1568	1.35 (2.82)		1603	0.84 (0.53)		1534	2.20 (3.00)		423	9.53 (18.92)	
- Yes	107	1.92 (2.54)		108	1.20 (0.84)		104	3.16 (3.12)		111	13.92 (20.87)	
Insurance status						***			***			**
- Uninsured	1012	1.42 (3.08)		1029	0.78 (0.53)		984	2.22 (3.28)		238	12.94 (21.37)	
- Insured	662	1.34 (2.33)		681	0.99 (0.58)		653	2.33 (2.57)		296	8.43 (17.45)	
Sex												
- Female	1074	1.43 (2.81)		1092	0.87 (0.58)		1052	2.32 (3.04)		363	10.17 (19.18)	
- Male	601	1.31 (2.80)		619	0.85 (0.52)		586	2.17 (2.97)		171	11.01 (19.93)	
Accompanier			***			***			***			**
- No	1320	1.18 (2.62)		1335	0.84 (0.55)		1290	2.03 (2.82)		405	8.80 (17.65)	
- Yes	355	2.15 (3.32)		376	0.95 (0.57)		348	3.12 (3.52)		129	15.59 (23.44)	
Facility Level			***			***			***			
- Dispensary	158	0.64 (1.50)		166	0.52 (0.44)		158	1.15 (1.70)		31	3.79 (10.93)	
- Health Centre	1124	1.46 (3.01)		1150	0.86 (0.52)		1101	2.34 (3.16)		327	11.29 (20.10)	
- Hospital	393	1.47 (2.58)		395	1.02 (0.64)		379	2.50 (2.92)		176	10.03 (19.09)	
Occupation						**						***
- Formal	210	1.11 (2.29)		227	0.81 (0.49)		207	1.92 (2.50)		51	1.99 (5.20)	
- Farmer	906	1.60 (3.24)		913	0.85 (0.58)		884	2.47 (3.44)		314	10.48 (19.05)	
- Self-employed	288	1.20 (2.26)		288	0.87 (0.58)		280	2.10 (2.54)		64	8.20 (17.08)	
- Other	266	1.10 (1.96)		278	0.94 (0.53)		262	2.03 (2.20)		100	14.70 (23.32)	

Note: * p <.05 ** p <.01 *** p <.001

For peer review only

Total non-medical costs averaged $2.26 (SD: $3.02), with an average direct cost of $1.39 (SD: $2.81) and indirect cost of $0.87 (SD: 0.56) (Table 2). The sub-analysis of self-reported forgone wages indicated an average opportunity cost of $21.88 (SD: $1180.83) (Table S2). NCD patients incurred significantly higher direct, indirect and total costs relative to other patients (Table 2, Figure 1). Furthermore, patients with comorbid NCDs spend more time seeking care compared to those with one condition and in turn bear higher opportunity costs. We also observed that those with insurance spend both more time travelling to the facility and more time at the facility, which could result from the fact that a higher proportion of insured people seek care at the hospital and higher facility levels have significantly higher access costs. Sensitivity analyses indicated that with the introduction of a 718 TZS minimum hourly wage, the increased value of time spent travelling and at the clinic leads to a 15.5% increase in the total non-medical cost of seeking care to $2.91 per care-seeking episode (Table S2).

Our analysis using mixed-effects multivariate linear regression revealed that having health insurance, hypertension, or multiple chronic conditions are associated with higher non-medical costs of care (as shown in Table 3). Specifically, patients with health insurance pay on average 14% more than uninsured patients, while those with hypertension or multiple chronic conditions pay 14% and 35% more, respectively. Higher education and seeking care at the closest facility to one's residence are associated with 11% lower non-medical costs. However, seeking care at a hospital (as opposed to a dispensary or health centre) is associated with a 67% increase in costs, and being accompanied to the facility is associated with a 39% increase in costs. In addition, we found a significant interaction effect for patients with hypertension who attend hospitals for their care, with costs being on average 35% higher than for other hospital outpatients (as shown in Table 4).

Table 3: Mixed-effects linear regression for total non-medical cost, including a random intercept for administrative district.

	Total Non-Medical Cost	95% CI
Health Insurance	0.137** (0.0504)	0.04 – 0.24
Hypertension	0.143* (0.0717)	0.002 – 0.28
Multiple Chronic Conditions	0.354*** (0.0998)	0.16 – 0.55
Sex (= male)	0.00384 (0.0487)	(0.09) – 0.10
Median Age	-0.0870 (0.0535)	(0.19) – 0.02
Higher education	-0.114** (0.0543)	(0.22) – (0.008)

For peer review only - http://bmjopen.bmj.com/site/about/guidelines.xhtml

Employed (last 12 months)	0.0353	(0.08) – 0.15
	(0.0561)	
Closest facility	-0.986***	(1.10) – (0.87)
	(0.0584)	
Accompanier	0.390***	0.28 – 0.50
	(0.0561)	
Hospital	0.673***	0.49 – 0.85
	(0.0915)	
Health Center	0.567***	0.41 – 0.73
	(0.0809)	
Hospital#Hypertension	0.352**	0.09 – 0.62
	(0.136)	
Constant	8.115***	7.92 – 8.31
	(0.0993)	
Observations	1,637	
Clusters (districts)	2	

Standard errors in parentheses
95% CI: negative numbers in parentheses
Note: * p <.05 ** p <.01 *** p <.001

Table 4: Negative binomial regression of the number of days in the past month that NCD patients' illness prevented from them working.

	Number of days missed work	95% CI
Health Insurance	-0.539***	(0.81) – (0.27)
	(0.139)	
Multiple Chronic Conditions	0.502***	0.21 – 0.80
	(0.150)	
Medicines prescribed	-0.782**	(1.30) – (0.27)
	(0.262)	
Medicines taken (last 7 days)	0.354	(0.04) – 0.75
	(0.201)	
Last time sought care		
- Within the last 6 months	-0.423	(0.93) – 0.08
	(0.259)	
- More than 6 months ago	-0.448	(0.90) – 0.003
	(0.230)	
Sex (=male)	0.0380	(0.24) – 0.32
	(0.144)	
Age	0.0284***	0.02 – 0.04
	(0.00573)	
Higher education	-0.629**	(1.03) – (0.22)
	(0.207)	
Occupation		

For peer review only

For peer review only - http://bmjopen.bmj.com/site/about/guidelines.xhtml

- Farmer	0.461 (0.326)	(0.18) – 1.10
- Self-employed	0.635 (0.363)	(0.08) – 1.35
- Other	0.857* (0.338)	0.19 – 1.52
Constant	0.190 (0.496)	(0.78) – 1.16
Observations	515	

Standard errors in parentheses
95% CI: negative numbers in parentheses
Note: * p <.05 ** p <.01 *** p <.001

For peer review only

We also observed that patients with multiple chronic conditions tend to miss more work days due to illness, while those with insurance or prescription medications tend to miss fewer. More highly educated individuals tend to miss fewer days, while older patients tend to miss more. However, we did not find any significant associations between treatment-seeking behaviour or medication adherence and absenteeism among patients with NCDs.

DISCUSSION

The results of this study reveal that patients still incur substantial direct and indirect non-medical costs when seeking health care, equivalent to 114% of the minimum daily wage[2]. These findings are consistent with previous research conducted in Tanzania and in other sub-Saharan African countries. One major contributor to these costs is the time required to reach the healthcare facilities. For instance, another study reported an average of 62 minutes to reach a hospital, compared to nearly 50 minutes in this study.[29] In a sample of 1407 patients requiring maternal and child health care (MCH), the average travel time was 30 minutes for a one-way trip, at an average cost of $0.41.[30] In addition, the reported average time spent at the clinic was almost an hour. These estimates are somewhat lower than our finding, which could be due to the greater and more decentralized availability of MCH services that do not require patients to travel as far. In fact, 70% of those seeking MCH care walked to the facility,[30] which is far higher than those in our sample.

[2] Considering a minimum hourly wage of $0.22 and nine working hours per day results in a daily wage of $1.98. The total non-medical cost was on average $2.26; yielding a cost to wage ratio of approximately 114%.

People living with HIV in rural Tanzania cross even larger distances, travelling an average of 2.81 hours in total and staying 2.32 hours at the clinic, but spend slightly less on transportation ($1.09) than in our sample.[11] The opportunity cost of illness was also lower ($3.79), with patients being ill for 16 hours per year on average, which is potentially due to the restriction of the sample to stable HIV patients. Specifically for NCD patients, annual transport costs amount to $14 in rural and $24 in urban areas, with average waiting times of 2 hours and 4 hours respectively.[22] In addition, patients reported an annual loss of income of $23 and $30 per year, respectively. Highly educated individuals incur lower time and travel costs in our sample, similar to other findings.[31] We suspect that this is a result of highly educated people being less likely to be engaged in strenuous physical labour and their proximity to health facilities, given that they tend to live in urban areas with high densities of facilities.

Our study revealed that health insurance status has contrasting effects on direct and indirect non-medical cost, with a negative association with travel cost and a positive association with time cost. The underlying reasons of this phenomenon are not entirely clear, but it is possible that individuals residing in urban areas, where health insurance is more common, also have easier access to healthcare facilities, thus incurring lower travel costs. However, urban facilities are often more crowded, leading to longer waiting times and higher time costs.[22]

The financial burden of seeking healthcare does not solely fall on patients, but also their informal caregivers. Our study shows that having an accompanier to bring patients to healthcare facilities is associated with significantly higher direct and indirect non-medical costs, even without accounting for the caregiver cost. Our data suggests that this is probably due to the most severely ill being significantly more likely to require accompaniment. Those accompanied by an informal caregiver are often unable to walk to the facility and may not receive care at their nearest facility, resulting in higher travel and time costs (Table 2). Additionally, the majority of participants reported their co-resident household members, such as their partner or child, as informal caregivers, indicating that these households face a double burden.

Patients with NCDs face such costs even more frequently than other patients, due to the need for monthly treatment and the frequent stockouts of essential medicines, which potentially explains why most survey participants' last doctor's visit occurred within the last month.[13, 32] Therefore, improving accessibility of NCD care at lower levels of care can reduce non-medical costs and improve the availability of medicines at these levels of care. This would allow patients to receive longer-term prescriptions, requiring fewer visits to the health care facility to refill medications. Decentralizing NCD services would not only provide more affordable care for NCD patients but would result in cost savings for insurers and the health system,[33] while generating additional revenue for lower-level facilities.

For peer review only - http://bmjopen.bmj.com/site/about/guidelines.xhtml

Furthermore, it is noteworthy that the opportunity cost associated with illness-related missed work surpasses the non-medical cost for accessing care, and is significantly higher for uninsured patients than insured ones, as they on average miss two more days of work per month. Absenteeism thus affects the uninsured more than the insured, knowing that direct OOP payments introduce a substantial financial barrier to accessing care.[34] However, our results suggest that engagement in care is associated with fewer missed work days. Moreover, retired, and hence older individuals, are most often unable to carry out their daily tasks, while those in formal employment seem to be the least impacted.

Our estimates of absenteeism due to chronic illness are higher than those reported in other studies. For instance, a study in Namibia found an average of only one day of sick leave over a 90-day period in employer records.[35] However, their study included both sick and non-sick employees, while our absenteeism data focused only on patients with chronic illnesses. Additionally, employer records may not capture the missed workdays of informal workers who make up the majority of Tanzania's workforce, particularly in the agriculture sector, where physically strenuous work is more likely to be impacted by chronic illness. To reduce the access and opportunity costs for NCD patients, it would be important to prioritize the provision of basic NCD services at lower levels of care and actively promote patients' engagement in care.

The study findings should be interpreted in the light of some limitations. The key limitation is the lack of reliable self-reported individual income estimates, which required us to rely upon the human capital method. In the supplementary materials, we provided an overview of the costs, as presented in Table 2, including the self-reported lost income due to seeking care on the day of the survey. These figures suggest that our estimates potentially underestimate the indirect non-medical cost and opportunity costs. However, participants are likely to overestimate their lost income, so we suspect that the actual cost lies somewhere in between. Other limitations are that travel, clinic time and number of missed days of work were self-reported and that the use of multiple modes of transportation was not considered when assessing travel costs. Additionally, exit surveys do not capture individuals with NCDs who do not seek healthcare at all, potentially hindered by the high cost burden. However, it does minimize recall bias related to the reported time and cost variables. While minimum wages have increased since the data collection for this study in 2020/2021, corresponding acceleration of inflation and fuel price increases may have negated any potential improvements to the affordability of the direct non-medical costs of seeking care.

In addition, we were unable to explore distress-financing strategies such as borrowing money or selling assets, nor could we stratify costs by socioeconomic status. Lastly, research has shown that the

For peer review only - http://bmjopen.bmj.com/site/about/guidelines.xhtml

direct and indirect costs of seeking care are higher during the rainy season,[36] as our data collection occurred during the dry season, we would expect the travel time to be higher during other times of the year.[37] Given that most patients were subsistence farmers, they potentially not only experience seasonality in their income, but also in their medical costs, which was not captured in this study.

Despite these limitations, this study sheds light on the challenges faced by Tanzanian NCD patients seeking care at health facilities, particularly for those without insurance. The study suggests that decentralizing the provision of NCD care from hospitals to health centres and dispensaries may be beneficial. This approach could help to reduce patients' non-medical and opportunity costs associated with travel and increase their engagement in care.

For peer review only

For peer review only - http://bmjopen.bmj.com/site/about/guidelines.xhtml

REFERENCES

1. Transforming our world: the 2030 Agenda for Sustainable Development, (2015).
2. Jan S, Laba TL, Essue BM, et al. Action to address the household economic burden of non-communicable diseases. Lancet. 2018;391(10134):2047-58.
3. Jakovljevic M, Jakab M, Gerdtham U, et al. Comparative financing analysis and political economy of noncommunicable diseases. Journal of Medical Economics. 2019;22(8):722-7.
4. Adinan J, Manongi R, Temu GA, et al. Preparedness of health facilities in managing hypertension & diabetes mellitus in Kilimanjaro, Tanzania: a cross sectional study. BMC Health Services Research. 2019;19(1).
5. Siddharthan T, Ramaiya K, Yonga G, et al. Noncommunicable Diseases In East Africa: Assessing The Gaps In Care And Identifying Opportunities For Improvement. Health Affairs. 2015;34(9):1506-13.
6. Bermudez-Tamayo C, Besançon S, Johri M, et al. Direct and indirect costs of diabetes mellitus in Mali: A case-control study. PLOS ONE. 2017;12(5):e0176128.
7. Mutyambizi C, Pavlova M, Chola L, et al. Cost of diabetes mellitus in Africa: a systematic review of existing literature. Globalization and Health. 2018;14(1):3.
8. Chimbindi N, Bor J, Newell M-L, et al. Time and Money: The True Costs of Health Care Utilization for Patients Receiving “Free” HIV/Tuberculosis Care and Treatment in Rural KwaZulu-Natal. JAIDS Journal of Acquired Immune Deficiency Syndromes. 2015;70(2):e52.
9. Mhalu G, Hella J, Mhimbira F, et al. Pathways and associated costs of care in patients with confirmed and presumptive tuberculosis in Tanzania: A cross-sectional study. BMJ Open. 2019;9(4):e025079.
10. Mudzengi D, Sweeney S, Hippner P, et al. The patient costs of care for those with TB and HIV: a cross-sectional study from South Africa. Health Policy Plan. 2017;32(suppl_4):iv48-iv56.
11. Okere NE, Corball L, Kereto D, et al. Patient-incurred costs in a differentiated service delivery club intervention compared to standard clinical care in Northwest Tanzania. J Int AIDS Soc. 2021;24(6):e25760.
12. Bommer C, Heesemann E, Sagalova V, et al. The global economic burden of diabetes in adults aged 20–79 years: a cost-of-illness study. The Lancet Diabetes & Endocrinology. 2017;5(6):423-30.
13. Macha J, Harris B, Garshong B, et al. Factors influencing the burden of health care financing and the distribution of health care benefits in Ghana, Tanzania and South Africa. Health Policy and Planning. 2012;27(suppl_1):i46-i54.
14. Varela C, Young S, Mkandawire N, et al. TRANSPORTATION BARRIERS TO ACCESS HEALTH CARE FOR SURGICAL CONDITIONS IN MALAWI a cross sectional nationwide household survey. BMC Public Health. 2019;19(1):264.
15. Kirigia JM, Sambo HB, Sambo LG, et al. Economic burden of diabetes mellitus in the WHO African region. BMC Int Health Hum Rights. 2009;9:6.
16. Wang Q, Brenner S, Kalmus O, et al. The economic burden of chronic non-communicable diseases in rural Malawi: an observational study. BMC Health Services Research. 2016;16(1):457.

For peer review only - http://bmjopen.bmj.com/site/about/guidelines.xhtml

17. Carls GS, Roebuck MC, Brennan TA, et al. Impact of medication adherence on absenteeism and short-term disability for five chronic diseases. J Occup Environ Med. 2012;54(7):792-805.
18. Jo C. Cost-of-illness studies: concepts, scopes, and methods. Clinical and Molecular Hepatology. 2014;20(4):327.
19. Shephard C, Hirst A, Buckland A, et al. Three Methods for Measuring Productivity Losses Due to Health Related Presenteeism in Economic Evaluations. Value in Health. 2016;19(7):A364-A5.
20. World Health O. WHO guide to identifying the economic consequences of disease and injury: World Health Organization; 2009 2009. 132 p.
21. Seuring T, Archangelidi O, Suhrcke M. The Economic Costs of Type 2 Diabetes: A Global Systematic Review. PharmacoEconomics. 2015;33(8):811-31.
22. Ngalesoni F, Ruhago G, Norheim OF, et al. Economic cost of primary prevention of cardiovascular diseases in Tanzania. Health Policy Plan. 2015;30(7):875-84.
23. Osetinsky B, Mhalu G, Mtenga S, et al. Care cascades for hypertension and diabetes: Cross-sectional evaluation of rural districts in Tanzania. PLOS Medicine. 2022;19(12):e1004140.
24. Government Notice No. 196 : Labour Institutions Wage Order. Gazette of the United Republic of Tanzania; 2013.
25. Bank of Tanzania. Previous Exchange Rates [Available from: https://www.bot.go.tz/ExchangeRate/previous_rates.]
26. Government Notice No. 687 : Labour Institutions Wage Order. United Republic of Tanzania; 2022.
27. StataCorp. Stata Stastical Software: Release 16. College Station, TX: StataCorp LLC; 2019.
28. Team RC. R: A language and evironment for statistical computing. Vienna, Austria: R Foundation for Statistical Computing; 2022.
29. Wong KLM, Brady OJ, Campbell OMR, et al. Too poor or too far? Partitioning the variability of hospital-based childbirth by poverty and travel time in Kenya, Malawi, Nigeria and Tanzania. International Journal for Equity in Health. 2020;19(1):15.
30. Binyaruka P, Borghi J. An equity analysis on the household costs of accessing and utilising maternal and child health care services in Tanzania. Health Economics Review. 2022;12(1).
31. Butt MD, Ong SC, Wahab MU, et al. Cost of Illness Analysis of Type 2 Diabetes Mellitus: The Findings from a Lower-Middle Income Country. International Journal of Environmental Research and Public Health. 2022;19(19):12611.
32. Kuwawenaruwa A, Wyss K, Wiedenmayer K, et al. The effects of medicines availability and stock-outs on household's utilization of healthcare services in Dodoma region, Tanzania. Health Policy Plan. 2020;35(3):323-33.
33. Hooley B, Osetinsky B, Tediosi F, editors. The cost of overutilizing hospitals for diabetes and hypertension care in Tanzania. iHEA; 2021 2021.
34. Huffman MD, Rao KD, Pichon-Riviere A, et al. A Cross-Sectional Study of the Microeconomic Impact of Cardiovascular Disease Hospitalization in Four Low- and Middle-Income Countries. PLoS One. 2011;6(6):e20821.
35. Guariguata L, de Beer I, Hough R, et al. Diabetes, HIV and other health determinants associated with absenteeism among formal sector workers in Namibia. BMC Public Health. 2012;12(1):44.

For peer review only

For peer review only - http://bmjopen.bmj.com/site/about/guidelines.xhtml

36. Su TT, Pokhrel S, Gbangou A, et al. Determinants of household health expenditure on western institutional health care. Eur J Health Econ. 2006;7(3):195-203.
37. Makanga PT, Schuurman N, Sacoor C, et al. Seasonal variation in geographical access to maternal health services in regions of southern Mozambique. International Journal of Health Geographics. 2017;16(1).

FIGURE LEGEND

Figure 1: Panel A, Total non-medical costs by health facility level and NCD status. Panel B, Total non-medical costs by health facility level and NCD status, including caregiver cost.

ACKNOWLEDGEMENTS

The authors wish to acknowledge the field workers of the Ifakara Health Institute for their data collection efforts, and the stakeholders and policymakers who attended dissemination workshops in Dodoma and Dar es Salaam whose discussions provided valuable input and context to this work.

COMPETING INTERESTS

The authors have no competing interests to declare.

FUNDING STATEMENT

This work was funded by the Swiss Programme for Research on Global Issues for Development (Grant number: 183760), a joint funding initiative by the Swiss Agency for Development and Cooperation (SDC) and the Swiss National Science Foundation (SNSF). The funders had no role in study design, data collection and analysis, decision to publish, or preparation of the manuscript.

For peer review only - http://bmjopen.bmj.com/site/about/guidelines.xhtml

For peer review only

For peer review only - http://bmjopen.bmj.com/site/about/guidelines.xhtml

For peer review only

For peer review only - http://bmjopen.bmj.com/site/about/guidelines.xhtml

For peer review only

For peer review only - http://bmjopen.bmj.com/site/about/guidelines.xhtml

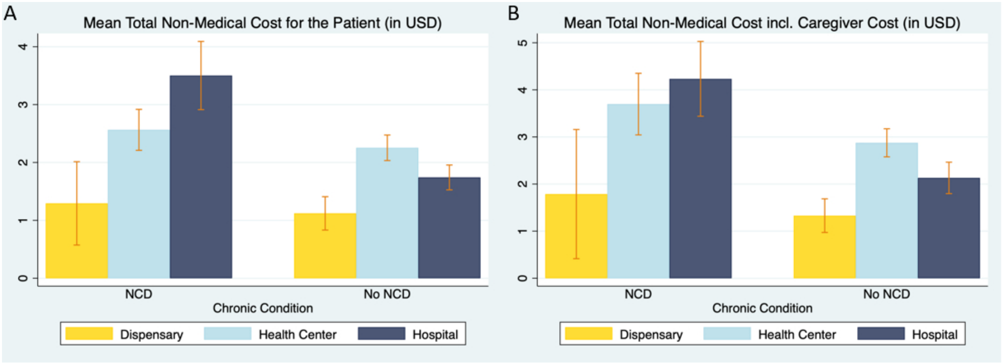

738x267mm (38 x 38 DPI)

SUPPLEMENTARY MATERIALS

Supplementary tables

Table S1: Descriptive statistics, disaggregated according to whether patients were accompanied by a caregiver.

		Accompanier		No Accompanier		
Variable	Obs.	Mean	Std. Dev.	Mean	Std. Dev.	p-value
Sex (male)	383	**.264**	.441	**.39**	.488	**<0.001**
Education level	.	.	.	.	.	0.083
Never attended school	383	.073	.261	.042	.202	
Some primary school	383	.123	.329	.122	.327	
**Completed primary school**	383	**.475**	.5	**.545**	.498	
Some secondary school	383	.047	.212	.035	.184	
Completed secondary	383	.219	.414	.188	.391	
Completed college education	383	.047	.212	.049	.216	
Completed university education	383	.016	.124	.018	.134	
Working (last 12 months)	383	.159	.366	.298	.458	**<0.001**
Occupation	.	.	.	.	.	**<0.001**
Public servant	383	.039	.194	.056	.229	
Private formal sector	383	.018	.134	.097	.296	
**Subsistence farmer**	383	**.527**	.5	**.532**	.499	
Large-scale farming	383	.005	.072	.004	.06	
Self-employed/small business	383	.146	.354	.171	.377	
Self-employed/large business	383	.003	.051	.004	.06	
Taking care of home and/or children	383	.102	.303	.033	.179	
Student	383	.055	.228	.036	.186	
Retired	383	.065	.247	.041	.198	
Other	383	.039	.194	.027	.162	
Marital status	.	.	.	.	.	**0.004**
**Married**	383	**.627**	.484	**.655**	.476	
Living with partner	383	.042	.2	.021	.144	
Divorced	383	.008	.088	.007	.085	
Separated	383	.021	.143	.054	.227	
Widowed	383	.151	.359	.107	.309	
Never married	383	.151	.359	.155	.362	
Age	383	43.919	19.048	44.195	16.202	0.78
Health insurance	383	.368	.483	.402	.491	0.22
Any SHP	383	.428	.495	.484	.5	0.055
Any chronic condition	383	.337	.473	.297	.457	0.13
Type of chronic condition						
- Hypertension	129	.861	.348	.790	.408	0.078
- Diabetes	129	.217	.414	.244	.430	0.52
- Chronic kidney disease	129	.031	.174	.007	.086	0.040
- Epilepsy						
- Asthma	129	.0	.0	.015	0.121	0.16
- HIV	129	.039	.194	.025	.155	0.40
- TB	129	.031	.174	.047	.212	0.44
- Other	129	.0	.0	.007	.086	0.33

For peer review only

	129	.093	.292	.069	.254	0.37
Multiple chronic conditions	129	.24	.429	.198	.399	0.30
Prevented from working						**<0.001**
Completely prevented	129	.442	.499	.533	.5	
Never prevented	129	.186	.391	.067	.25	
Sometimes prevented	129	.372	.485	.4	.491	
Days missed work	129	7.69	11.565	4.341	8.708	**<0.001**
Facility level	.	.	.	.	.	**0.003**
Dispensary	383	.081	.273	.099	.299	
**Health centre**	383	**.742**	.438	**.651**	.477	
Hospital	383	.178	.383	.25	.433	
Closest facility visited	383	.791	.407	.888	.316	**<0.001**
Normal facility visited	383	.749	.434	.832	.374	**<0.001**
Transportation mode	.	.	.	.	.	**<0.001**
**Walk**	383	**.295**	.457	**.494**	.5	
Bicycle	383	.063	.243	.071	.257	
Your own motorbike	383	.078	.269	.033	.179	
Motorbike taxi	383	.36	.481	.28	.449	
Your own car	383	.06	.238	.018	.134	
Bus	383	.084	.277	.084	.278	
Bajaj	383	.037	.188	.015	.123	
A friend or family member brought me	383	.01	.102	.001	.038	
Other	383	.013	.114	.001	.038	
Travel time (return) (mins)	381	117.85	102.23	93.28	87.30	**<0.001**
Clinic time (mins)	378	134.45	103.06	129.94	104.50	0.46

For peer review only

Table S2: Direct non-medical and indirect non-medical costs of care-seeking, estimated using both the human capital method and self-reported lost earnings

	**Direct Non-Medical Cost**			**Indirect Non-Medical Cost**			**Total Non-Medical Cost**			**Estimated Lost Income (NCD patients)**		
	N	Mean (SD)	p-value	N	Mean (SD)	p-value	N	Mean (SD)	p-value	N	Mean (SD)	p-value
All	1675	1.39 (2.81)		1617	1.51 (2.13)		1550	2.93 (3.67)		498	21.88 (180.83)	
Chronic Condition			***			*			**			
No	1165	1.22 (2.68)		1117	1.48 (2.04)		1072	2.76 (3.50)			.	
Yes	510	1.76 (3.05)		500	1.58 (2.30)		478	3.32 (3.99)			.	
Multimorbidity			***						**			**
No	1568	1.35 (2.82)		1518	1.50 (2.14)		1454	2.89 (3.66)		399	22.70 (201.31)	
Yes	107	1.92 (2.54)		99	1.67 (1.91)		96	3.60 (3.77)		99	18.56 (35.14)	
Insurance status						***			***			*
Uninsured	1012	1.42 (3.08)		960	1.32 (1.72)		920	2.80 (3.72)		219	14.56 (24.80)	
Insured	662	1.34 (2.33)		656	1.79 (2.59)		629	3.12 (3.59)		279	27.62 (240.62)	
Sex						***						
Female	1074	1.43 (2.81)		1055	1.32 (1.80)		1017	2.76 (3.47)		341	11.87 (23.61)	
Male	601	1.31 (2.80)		562	1.86 (2.60)		533	3.26 (4.01)		157	43.61 (319.79)	
Accompanier			***						***			*
No	1320	1.18 (2.62)		1251	1.57 (2.29)		1211	2.79 (3.64)		376	23.87 (207.70)	
Yes	355	2.15 (3.32)		366	1.28 (1.40)		339	3.44 (3.72)		122	15.74 (23.61)	
Facility Level			***			***			***			
Dispensary	158	0.64 (1.50)		159	1.22 (2.29)		151	1.91 (2.99)		31	6.15 (17.39)	
Health Centre	1124	1.46 (3.01)		1122	1.45 (2.00)		1075	2.92 (3.66)		314	13.77 (26.53)	
Hospital	393	1.47 (2.58)		336	1.84 (2.40)		324	3.44 (3.89)		153	41.70 (323.77)	
Occupation						***			***			**
Formal	210	1.11 (2.29)		177	2.96 (3.38)		161	4.15 (4.22)		39	122.78 (639.09)	
Farmer	906	1.60 (3.24)		882	1.17 (1.24)		855	2.81 (3.59)		299	12.20 (25.25)	
Self-employed	288	1.20 (2.26)		280	1.83 (2.88)		272	3.09 (3.92)		58	10.92 (22.66)	
Other	266	1.10 (1.96)		273	1.33 (2.03)		257	2.44 (3.10)		97	16.87 (27.29)	

For peer review only

Table S3: Sensitivity analysis of Table 2 with new minimum hourly wage introduced from January 2023 (718 TZS ~ 0-30 USD)

	**Direct Non-Medical Cost**			**Indirect Non-Medical Cost**			**Total Non-Medical Cost**			**Estimated Lost Income (NCD patients)**		
	N	Mean (SD)	p-value	N	Mean (SD)	p-value	N	Mean (SD)	p-value	N	Mean (SD)	p-value
All	1675	1.39 (2.81)		1711	1.21 (0.78)		1638	2.61 (3.11)		534	14.62 (27.17)	
Chronic Condition			***			***			***			
No	1165	1.22 (2.68)		1189	1.12 (0.70)		1140	2.35 (2.89)			.	
Yes	510	1.76 (3.05)		522	1.41 (0.92)		498	3.20 (3.51)			.	
Multimorbidity			***			***			***			***
No	1568	1.35 (2.82)		1603	1.18 (0.74)		1534	2.54 (3.08)		423	13.34 (26.49)	
Yes	107	1.92 (2.54)		108	1.68 (1.17)		104	3.65 (3.37)		111	19.48 (29.22)	
Insurance status						***			***			**
Uninsured	1012	1.42 (3.08)		1029	1.10 (0.74)		984	2.53 (3.37)		238	18.11 (29.91)	
Insured	662	1.34 (2.33)		681	1.39 (0.82)		653	2.73 (2.69)		296	11.80 (24.44)	
Sex												
Female	1074	1.43 (2.81)		1092	1.22 (0.81)		1052	2.67 (3.15)		363	14.24 (26.85)	
Male	601	1.31 (2.80)		619	1.19 (0.73)		586	2.51 (3.05)		171	15.42 (27.90)	
Accompanier			***			***			***			**
No	1320	1.18 (2.62)		1335	1.18 (0.78)		1290	2.37 (2.92)		405	12.32 (24.71)	
Yes	355	2.15 (3.32)		376	1.33 (0.80)		348	3.50 (3.62)		129	21.82 (32.82)	
Facility Level			***			***			***			
Dispensary	158	0.64 (1.50)		166	0.73 (0.61)		158	1.36 (1.81)		31	5.31 (15.30)	
Health Centre	1124	1.46 (3.01)		1150	1.20 (0.73)		1101	2.68 (3.23)		327	15.80 (28.14)	
Hospital	393	1.47 (2.58)		395	1.43 (0.90)		379	2.92 (3.07)		176	14.05 (26.73)	
Occupation						**						***
Formal	210	1.11 (2.29)		227	1.14 (0.69)		207	2.25 (2.61)		51	2.78 (7.28)	
Farmer	906	1.60 (3.24)		913	1.20 (0.81)		884	2.81 (3.53)		314	14.67 (26.68)	
Self-employed	288	1.20 (2.26)		288	1.22 (0.81)		280	2.45 (2.67)		64	11.49 (23.91)	
Other	266	1.10 (1.96)		278	1.31 (0.74)		262	2.40 (2.32)		100	20.58 (32.65)	

For peer review only

Table S4: Sensitivity analysis of mixed-effects linear regression for total non-medical cost, including a random intercept for administrative district

	Total Non-Medical Cost	95% CI
Health Insurance	0.136**<br>(0.046)	0.05 – 0.23
Hypertension	0.133*<br>(0.065)	0.005 – 0.26
Multiple Chronic Conditions	0.32***<br>(0.091)	0.14 – 0.50
Sex (= male)	0.01<br>(0.044)	(0.08) – 0.10
Median Age	-0.077<br>(0.049)	(0.17) – 0.02
Higher education	-0.115**<br>(0.049)	(0.21) – (0.018)
Employed (last 12 months)	0.027<br>(0.0561)	(0.07) – 0.13
Closest facility	-0.898***<br>(0.0584)	(1.00) – (0.79)
Accompanier	0.390***<br>(0.051)	0.25 – 0.45
Hospital	0.649***<br>(0.083)	0.49 – 0.81
Health Center	0.546***<br>(0.074)	0.40 – 0.69
Hospital#Hypertension	0.321**<br>(0.123)	0.08 – 0.56
Constant	8.312***	8.13 – 8.49

For peer review only

	(0.09)
Observations	1,637
Clusters (districts)	2

Standard errors in parentheses
95% CI: negative numbers in parentheses
Note: * p <.05 ** p <.01 *** p <.001

For peer review only

Author reflexivity statement

1. How does this study address local research and policy priorities?

This study directly addresses the as-yet underappreciated issue of indirect costs of accessing healthcare in Tanzania, which disproportionately affects Tanzania's majority rural population. This study brings attention to this local issue, and makes recommendations for improving the equity of access to NCD care in Tanzania.

2. How were local researchers involved in study design?

Local researchers KT and GM were involved in the design of the greater research project within which this study is nested, in addition to having been involved in the design of the methodology for the present study. They both have rich experience in international research partnerships, completing and having completed their doctorate at the University of Basel, respectively. They represent a gender- and seniority-balanced local research team composition.

3. How has funding been used to support the local research team?

This study was nested within the broader "Health systems governance for an inclusive and sustainable social health protection in Ghana and Tanzania" study, funded by the Swiss programmes for research on global issues for development that was jointly obtained by Switzerland- and Tanzania-based co-PIs, Prof. Dr. Fabrizio Tediosi and Dr. Sally Mtenga respectively. Throughout the 6-year lifespan of this project, this grant supported equal numbers of Switzerland- and Tanzania-based doctoral students and researchers.

4. How are research staff who conducted data collection acknowledged?

This study recruited research assistants to perform data collection, who are appropriate credited in the acknowledgements section.

5. Do all members of the research partnership have access to study data?

All members of the partnership have access to the study data.

6. How was data used to develop analytical skills within the partnership?

Both Switzerland- and Tanzania-based collaborated on the design of data collection tools, further developing their skills in programming ODK forms. Both Tanzania- and Switzerland-based co-authors had the opportunity to follow courses at the University of Basel to develop the analytical and data analysis skills, while directly working with the data stemming from this study.

7. How have research partners collaborated in interpreting study data?

Authors BH and KT worked together on the cleaning and initial interpretation of study data.

8. How were research partners supported to develop writing skills?

For peer review only

The first authors of this manuscript are junior academics, who received support from senior academic FT throughout the writing process.

9. How will research products be shared to address local needs?

This work has been directly disseminate via two stakeholder panel discussions and presentations with stakeholders and policymakers from the Ministry of Health, Community Development, Elderly and Children, the President's Office for Regional Administration and Local Government, and the Tanzanian National Health Insurance Fund. Both events were attended by local journalists and media, which helped ensure a wider local dissemination of findings.

10. How is the leadership, contribution and ownership of this work by LMIC researchers recognised within the authorship?

Authors RM and NO worked as part of the senior authorship team in developing this manuscript, and their contribution has been recognised as joint first and joint last authors respectively. We have specifically included researchers based in the global south (RM and NO) within the senior authorship team as joint first and joint last authors. We acknowledge, however, that the authorship team is predominantly based in high-income countries. The primary reason for this is that the initiative has been driven from the perspective of journal editors with insight on how to develop transparent and implementable guidelines to assess manuscript submissions from international partnerships.

11. **How have early career researchers across the partnership been included within the authorship team?**

We have included early career researchers (CK, SS & MS) within the authorship team. They attended all the workshops, contributed to the literature review and evidence synthesis and to development of the consensus recommendations. We acknowledge that they are based in high-income countries.

12. How has gender balance been addressed within the authorship?

Three co-authors are men (BH, KT and FT) and two co-authors women (AV, GM).

13. How has the project contributed to training of LMIC researchers?

All researchers based in Tanzania (KT and GM) received or are receiving doctoral training and education in Switzerland, at the University of Basel, financed by the Research for development grant described in point number three.

14. How has the project contributed to improvements in local infrastructure?

This project did not directly contribute to improving local infrastructure.

15. What safeguarding procedures were used to protect local study participants and researchers?

For peer review only

All participants provided informed consent prior to participation. Research protocols were critically reviewed and approved by the institutional review board of the Ifakara Health Institute and the National Institute for Medical Research of Tanzania.

For peer review only

For peer review only

For peer review only

STROBE Statement—Checklist of items that should be included in reports of ***cross-sectional studies***

	Item No	Recommendation
**Title and abstract**	1	(*a*) Indicate the study's design with a commonly used term in the title or the abstract
		(*b*) Provide in the abstract an informative and balanced summary of what was done and what was found
**Introduction**		
Background/rationale	2	Explain the scientific background and rationale for the investigation being reported
Objectives	3	State specific objectives, including any prespecified hypotheses
**Methods**		
Study design	4	Present key elements of study design early in the paper
Setting	5	Describe the setting, locations, and relevant dates, including periods of recruitment, exposure, follow-up, and data collection
Participants	6	(*a*) Give the eligibility criteria, and the sources and methods of selection of participants
Variables	7	Clearly define all outcomes, exposures, predictors, potential confounders, and effect modifiers. Give diagnostic criteria, if applicable
Data sources/ measurement	8*	For each variable of interest, give sources of data and details of methods of assessment (measurement). Describe comparability of assessment methods if there is more than one group
Bias	9	Describe any efforts to address potential sources of bias
Study size	10	Explain how the study size was arrived at
Quantitative variables	11	Explain how quantitative variables were handled in the analyses. If applicable, describe which groupings were chosen and why
Statistical methods	12	(*a*) Describe all statistical methods, including those used to control for confounding
		(*b*) Describe any methods used to examine subgroups and interactions
		(*c*) Explain how missing data were addressed
		(*d*) If applicable, describe analytical methods taking account of sampling strategy
		(*e*) Describe any sensitivity analyses
**Results**		
Participants	13*	(a) Report numbers of individuals at each stage of study—eg numbers potentially eligible, examined for eligibility, confirmed eligible, included in the study, completing follow-up, and analysed
		(b) Give reasons for non-participation at each stage
		(c) Consider use of a flow diagram
Descriptive data	14*	(a) Give characteristics of study participants (eg demographic, clinical, social) and information on exposures and potential confounders
		(b) Indicate number of participants with missing data for each variable of interest
Outcome data	15*	Report numbers of outcome events or summary measures
Main results	16	(*a*) Give unadjusted estimates and, if applicable, confounder-adjusted estimates and their precision (eg, 95% confidence interval). Make clear which confounders were adjusted for and why they were included
		(*b*) Report category boundaries when continuous variables were categorized
		(*c*) If relevant, consider translating estimates of relative risk into absolute risk for a meaningful time period
Other analyses	17	Report other analyses done—eg analyses of subgroups and interactions, and sensitivity analyses

For peer review only - http://bmjopen.bmj.com/site/about/guidelines.xhtml
For peer review only

**Discussion**		
Key results	18	Summarise key results with reference to study objectives
Limitations	19	Discuss limitations of the study, taking into account sources of potential bias or imprecision. Discuss both direction and magnitude of any potential bias
Interpretation	20	Give a cautious overall interpretation of results considering objectives, limitations, multiplicity of analyses, results from similar studies, and other relevant evidence
Generalisability	21	Discuss the generalisability (external validity) of the study results
**Other information**		
Funding	22	Give the source of funding and the role of the funders for the present study and, if applicable, for the original study on which the present article is based

*Give information separately for exposed and unexposed groups.

Note: An Explanation and Elaboration article discusses each checklist item and gives methodological background and published examples of transparent reporting. The STROBE checklist is best used in conjunction with this article (freely available on the Web sites of PLoS Medicine at http://www.plosmedicine.org/, Annals of Internal Medicine at http://www.annals.org/, and Epidemiology at http://www.epidem.com/). Information on the STROBE Initiative is available at www.strobe-statement.org.

For peer review only

BMJ Open

A cross-sectional study of the burden and determinants of non-medical and opportunity costs of accessing chronic disease care in rural Tanzania

Journal:	*BMJ Open*
Manuscript ID	bmjopen-2023-080466.R1
Article Type:	Original research
Date Submitted by the Author:	03-Jan-2024

Complete List of Authors:	Verjans, Anna; Swiss Tropical and Public Health Institute; University of Basel<br>Hooley, Brady; Swiss Tropical and Public Health Institute; University of Basel, Faculty of Medicine<br>Tani, Kassimu; Swiss Tropical and Public Health Institute; University of Basel<br>Mhalu, Grace; Ifakara Health Institute<br>Tediosi, Fabrizio; Swiss Tropical and Public Health Institute; University of Basel

**Primary Subject Heading**:	Public health
Secondary Subject Heading:	Global health, Health economics, Health services research
Keywords:	Chronic Disease, HEALTH ECONOMICS, Health Services Accessibility, Health policy < HEALTH SERVICES ADMINISTRATION & MANAGEMENT, Health Services for the Aged

SCHOLARONE™
Manuscripts

I, the Submitting Author has the right to grant and does grant on behalf of all authors of the Work (as defined in the below author licence), an exclusive licence and/or a non-exclusive licence for contributions from authors who are: i) UK Crown employees; ii) where BMJ has agreed a CC-BY licence shall apply, and/or iii) in accordance with the terms applicable for US Federal Government officers or employees acting as part of their official duties; on a worldwide, perpetual, irrevocable, royalty-free basis to BMJ Publishing Group Ltd ("BMJ") its licensees and where the relevant Journal is co-owned by BMJ to the co-owners of the Journal, to publish the Work in this journal and any other BMJ products and to exploit all rights, as set out in our licence.

The Submitting Author accepts and understands that any supply made under these terms is made by BMJ to the Submitting Author unless you are acting as an employee on behalf of your employer or a postgraduate student of an affiliated institution which is paying any applicable article publishing charge ("APC") for Open Access articles. Where the Submitting Author wishes to make the Work available on an Open Access basis (and intends to pay the relevant APC), the terms of reuse of such Open Access shall be governed by a Creative Commons licence – details of these licences and which Creative Commons licence will apply to this Work are set out in our licence referred to above.

Other than as permitted in any relevant BMJ Author's Self Archiving Policies, I confirm this Work has not been accepted for publication elsewhere, is not being considered for publication elsewhere and does not duplicate material already published. I confirm all authors consent to publication of this Work and authorise the granting of this licence.

For peer review only

A cross-sectional study of the burden and determinants of non-medical and opportunity costs of accessing chronic disease care in rural Tanzania

For peer review only

For peer review only - http://bmjopen.bmj.com/site/about/guidelines.xhtml

Key words: Chronic diseases, Outpatient care, Tanzania, Health service provision, Indirect health expenditure, Self-referral

ABSTRACT

Objectives: Countries in sub-Saharan Africa are seeking to improve access to healthcare through health insurance. However, patients still bear non-medical costs and opportunity costs in terms of lost work days. The burden of these costs is particularly high for people with chronic diseases (CDs) who require regular healthcare. This study quantified the non-medical and opportunity costs faced by chronic disease patients in Tanzania and identified factors that drive these costs.

Methods: From November 2020 to January 2021, we conducted a cross-sectional patient survey at 35 healthcare facilities in rural Tanzania. Using the human capital approach to value the non-medical cost of seeking healthcare, we employed multilevel linear regression to analyse the impact of CDs and health insurance on non-medical costs and negative binomial regression to investigate the factors associated with opportunity costs of illness among patients with CDs.

Results: Among 1748 patients surveyed, 534 had at least one CD, 20% of which had comorbidities. Patients with CDs incurred significantly higher non-medical costs than other patients, with an average of $2.79 (SD: 3.36) compared to $2.03 (SD: 2.82). In addition, they incur a monthly illness-related opportunity cost of $10.19 ($0 - $59.34). Factors associated with higher non-medical costs included multimorbidities, hypertension, health insurance, and seeking care at hospitals rather than other facilities. Patients seeking hypertension care at hospitals experienced 35% higher costs compared to those visiting other facilities. Additionally, patients with comorbidities, older age, less education, and those requiring medication more frequently lost workdays.

Conclusion: Outpatient care in Tanzania imposes considerable non-medical costs, particularly for people with CDs, besides illness-related opportunity costs. Despite having health insurance, patients with CDs who seek outpatient care in hospitals face higher financial burdens than other patients. Policies to improve the availability and quality of CD care in dispensaries and health centres could reduce these costs.

For peer review only

For peer review only - http://bmjopen.bmj.com/site/about/guidelines.xhtml

Strengths and limitations of this study

- This study includes data from 35 facilities – all health centres and hospitals and a sample of dispensaries – in the Same and Kilombero districts of Tanzania, and a large sample size, which strengthen the robustness of the results.
- We employed exit surveys, which minimize the recall bias related to the reported time and transportation cost variables, but does not capture those who might forgo healthcare because of financial barriers.
- The main limitation of this study is the lack of reliable self-reported individual income estimates, which is why we adopted the minimum daily wage instead.
- However, the advantage of using the minimum wage to value patients' time is that it equally values their time, instead of assigning a higher value to the time of patients with higher salaries.

For peer review only - http://bmjopen.bmj.com/site/about/guidelines.xhtml

INTRODUCTION

Universal health coverage aims to ensure "that people have access to the healthcare they need without suffering financial hardship".(1) Country-level policies have so far focused on reducing the direct medical out-of-pocket (OOP) cost of healthcare services and drugs, without addressing the non-medical costs of illness such as loss of income and the cost of travelling to access healthcare.(2) Utilizing healthcare induces costs that extend beyond the medical costs covered by health insurance and involves transportation and income losses for both patients and any accompanying caregivers.(2)

This narrow focus of social health protection and service provision is particularly problematic for health systems in low- and middle-income countries (LMICs), which are still mainly focused on the management of communicable diseases where the household economic burden associated with illness is more readily characterised by discrete, yet still potentially catastrophic disease events.(3, 4) On the other hand, despite mounting evidence of the increased burden of non-communicable diseases (NCDs) in LMICs, care for chronic diseases (CDs) remains centralized at hospitals and many medicines for the treatment of these conditions are not readily available at primary and secondary care facilities.(5) As a result, the financial burden of seeking chronic disease care tends to be greater than that of seeking acute care, in that chronic disease patients may need to utilise healthcare services more frequently and seek centralised care at hospitals in order to ensure that they receive the care they require.(6, 7)

The issue of burdensome non-medical healthcare costs has been studied in sub-Saharan Africa (SSA), particularly in the context of human immunodeficiency virus (HIV) and tuberculosis (TB) care.(8-10) As with NCDs, the chronic nature of HIV and TB management requires patients to visit healthcare facilities and renew prescriptions on a 1-3 month basis, meaning that patients must regularly pay for transport to the clinic and spend time accessing care that could have otherwise been spent working.(9, 10) The accumulation of travel costs and productivity loss associated with illness and care seeking can therefore surpass the direct cost of care, representing 55% of costs for HIV infections and 71% of costs for HIV/TB coinfections.(10) However, the introduction of a decentralised community-based HIV care program in Tanzania substantially reduced both the indirect and direct costs of accessing HIV care, indicating that such an intervention or policy implementation could yield promising results for reducing the indirect costs of care seeking for people living with chronic conditions.(11)

While research on the non-medical and opportunity costs of seeking NCD care in SSA is more limited, past work has indicated that these costs can be substantial for some patients, and some of

For peer review only - http://bmjopen.bmj.com/site/about/guidelines.xhtml

them even completely forgo seeking care in order to avoid the impoverishing effect of direct medical or non-medical costs of care.(6, 12-15) In Mali, the indirect costs of diabetes mellitus were estimated to make up 61% of the total costs,(6) while in rural Malawi the direct costs of NCDs formed the largest cost element, with 56.8% of the total.(16) While not directly comparable, their findings demonstrate a substantial economic burden on patients.

Non-medical costs form a barrier that can therefore affect uptake and adherence to treatment, which for chronic conditions can contribute to severe long-term effects. In this regard, prevention and early treatment of NCDs can avoid the need for more expensive treatment procedures in the future, caused by more severe illness, which usually also requires more frequent follow-up. For instance, a study found higher transportation costs and longer waiting times for anti-retroviral therapy (ART) compared to pre-ART patients in rural South Africa.(8) Besides early onset of treatment, absenteeism and short-term disability among chronic disease patients can also be reduced by improving medication adherence.(17, 18) This highlights the potential economic gain of implementing alternative care models that lower the financial burden for NCD patients by avoiding future opportunity costs.

The human capital method (HCM) and friction cost method are two of the most commonly used methods for measuring opportunity costs. The former measures opportunity costs in terms of the value of lost income, including future productivity losses, while the latter only considers these losses up until the market finds a replacement.(19) The HCM takes a patient perspective and is often preferred over the friction cost method since it is better able to account for presenteeism, although it is sometimes criticized for overestimating the costs as it assumes that the duration of absence from work fully corresponds to the market value of those lost days.(20) However, the dependence of agricultural economies on seasonality means that sick days do not necessarily translate into lost working days.(21) Moreover, to compensate for lost working time and income, households often adopt various 'distress financing' strategies, such as borrowing money or selling assets, especially in informal economies.(21) Income losses in the agricultural sector are sometimes approximated by the minimum daily wage of the country or by taking the GNI per capita and transforming it into a daily value,(6, 15, 22-24) the latter of which may inflate opportunity costs.(25, 26) The former method aims to address the lack of equity of the HCM by valuing everyone's time equally.

Previous work in Tanzania showed that people with cardiovascular risk factors incur substantial non-medical and opportunity costs, yet there is little evidence regarding the actual non-medical costs of seeking NCD care in Tanzania.(27) Considering the potential burden of non-medical costs of seeking healthcare and opportunity costs of losing workdays due to illness, this study surveyed outpatients

For peer review only

For peer review only - http://bmjopen.bmj.com/site/about/guidelines.xhtml

from 35 health facilities in two rural districts in order to estimate these costs in the rural Tanzanian context.

METHODS

This study is part of the Chronic Conditions Household and Exit Survey in Tanzania (CHEST), a cross-sectional outpatient and household survey in the rural Same and Kilombero districts conducted between November 2020 and January 2021. This survey recruited 784 household members and 1748 [1] outpatients above the age of 18. This study used only outpatient data on the reason for their health facility visit, time and cost of transport to the facility, time spent at the clinic and what they expect they would have earned if they had not sought care that day. The sampling strategy and full details of the CHEST survey have been described in previous work.(28)

The patient health facility exit survey was administered to adult patients at all tertiary and secondary health facilities in both districts and a matched sample of 8 dispensaries in each of the Kilombero and Same districts. As there are 8 health centres in each of these districts, the dispensary sampling was matched by randomly selecting one dispensary from each ward containing a health centre. All adult patients attending the outpatient clinic on the day of the survey were eligible for recruitment.

Outpatient clinics in hospitals and health centres typically designate one day a week as an 'NCD day', when a medical officer is assigned to be available to provide outpatient NCD care.(28) Therefore, for each sampled hospital and health centre, the exit surveys were conducted on an NCD day at the health facility to ensure that a sufficient number of participants with NCDs would receive the questionnaire, and on a non-NCD day at the clinic to also collect a more typical sample of people seeking outpatient care.

Rather than random or systematic random sampling of participants, this study used the more operationally efficient method of selecting and recruiting participants based on the order in which they entered the consultation room. This is demonstrated to be easier to implement than random sampling approaches, and it minimises the bias in consultation length associated with sampling patients as they leave the consultation room.(29)

For peer review only

[1] We calculated the sample size based on the hypertension and diabetes prevalence for rural Tanzanian adults, and population estimates for the two districts. A modified Cochran sample size calculation (power: 0.80, significance level: 5%) resulted in a minimum sample size of 202 patients per district.(28)

For peer review only - http://bmjopen.bmj.com/site/about/guidelines.xhtml

The outcome of interest was the total non-medical cost of seeking healthcare services, comprising both direct and indirect non-medical costs. In this case, direct non-medical cost included transportation fare while the indirect non-medical costs included the opportunity cost associated with travel and clinic time. We excluded those individuals with missing data for the travel cost or travel or clinic time from the analysis, resulting in 1638 individuals with full cost data. We estimated the indirect non-medical cost using the HCM, utilizing the national minimum hourly wage for the agricultural sector (512.82 TZS ~ 0.22 USD), the largest occupational group in our sample.(30) However, some salaried participants reported lost income, so we performed a sub-analysis of this data, while imputing human capital-based opportunity costs for those participants who did not provide estimates for their forgone wages (see supplementary materials). All costs were converted to 2020 US Dollars (1 USD = 2277 TZS).(31) For survey participants who claimed to have a chronic condition, we additionally estimated the cost of lost work over the past month due to illness, based on the number of days they reported being unable to work.

We used a mixed-effects multivariate linear regression to investigate the association between non-medical costs and insurance status and chronic disease diagnoses, while controlling for various demographic factors such as gender, age, education, occupation, accompaniment by a caregiver, level of healthcare facility, and residential proximity to the facility. We included a random intercept term to account for between-district variability, and log transformed non-medical costs in order to maintain the assumptions of linear regression. Additionally, we included an interaction term to examine the impact of hospital care on patients with hypertension.

On January 1st, 2023, the Government of Tanzania introduced a new wage order, increasing the hourly wage of agriculture workers by approximately 40%, from 512.82 TZS to 718 TZS.(30, 32). We therefore performed a sensitivity analysis to investigate the impact of higher wages on indirect health expenditure by using the new minimum wage in human capital method estimates of opportunity costs.

To evaluate the impact of reduced work capacity on patients with CDs, we used negative binomial regression[2] to determine the association between engagement in care and the number of days missed from work over the last 30 days. The predictor variables of interest included the prescription of medication during the last visit, medication adherence over the last week, and the presence of multiple chronic conditions, with controls for age, gender, education, and occupation. We used both STATA SE 16.1 and R (v.4.1.3) for analysis.(33, 34)

For peer review only

[2] Poisson regression models were tested against negative binomial models using likelihood ratio tests. Given the significant overdispersion, the negative binomial model was preferred, which is in line with analysis standards for absenteeism data.

Ethical clearance

This studied obtained ethical clearance from the Ifakara Health Institute (IHI) Institutional Review Board (IHI/IRB/EXT/No:35–2020) and the Tanzanian National Institute for Medical Research (NIMR/HQ/R.8a/Vol.IX/3518).

Patient and public involvement statement

We first involved the public by engaging with healthcare providers prior to the design of this study, where informal conversations with providers at rural hospitals and health centres indicated that few NCD services are available at dispensaries and health centres and that patients with NCDs must frequently be referred or self-refer over substantial distances for relatively basic NCD services and prescription medicines. These providers lamented that the need to seek chronic disease care in hospitals forms both a substantial barrier to care that prevents would-be patients from being retained in care in a timely manner, and that those who are retained in care face substantial non-medical costs.

The findings of this study were directly presented to Tanzanian stakeholders and policymakers via workshops and presentations in Dodoma and Dar es Salaam, where we engaged with attendees from the Ministry of Health, National Health Insurance Fund, Improved Community Health Fund and President's Office for Regional Administration and Local Government.

RESULTS

We recruited 1748 outpatients, of which 63.73% were women, 64.87% married, 52.97% completed primary school, 53.09% subsistence farmers and a mean age of 44 years (Table 1). Forty percent were health insurance beneficiaries and about 30% had a chronic condition. Our analysis focused on chronic conditions, of which the majority are NCDs, with hypertension being the most commonly reported condition (81%). Approximately 21% of patients with CDs had more than one chronic condition. Patients with CDs lost an average of five working days due to their illness during the month before the survey, with almost 10% completely unable to work.

Table 1: Descriptive statistics of the total sample

Variable	N	Mean	SD
Sex (male)	1748	36.3%	48.1%
Education level	.	.	.
Never attended school	1748	4.9%	21.6%
Some primary school	1748	12.2%	32.7%
Completed primary school	1748	53%	49.9%

For peer review only

For peer review only - http://bmjopen.bmj.com/site/about/guidelines.xhtml

Some secondary school	1748	3.8%	19.1%
Completed secondary	1748	19.5%	39.6%
Completed college education	1748	4.9%	21.5%
Completed university education	1748	1.8%	13.2%
Working (last 12 months)	1748	26.8%	44.3%
Occupation	.	.	.
Public servant	1748	5.2%	22.2%
Private formal sector	1748	8%	27.1%
Subsistence farmer	1748	53.1%	49.9%
Large-scale farming	1748	0.4%	6.3%
Self-employed/small business	1748	16.6%	37.2%
Self-employed/large business	1748	0.3%	5.9%
Taking care of home and/or children	1748	4.8%	21.4%
Student	1748	4%	19.6%
Retired	1748	4.6%	21%
Other	1748	3%	17%
Marital status	.	.	.
Married	1748	64.9%	47.8%
Living with partner	1748	2.6%	15.8%
Divorced	1748	0.7%	8.6%
Separated	1748	4.7%	21.2%
Widowed	1748	11.7%	32.1%
Never married	1748	15.4%	36.1%
Age	1748	44.134	16.861
Health insurance	1747	39.5%	48.9%
Any SHP	1748	47.1%	49.9%
Any chronic condition	1748	30.5%	46.1%
Type of chronic condition			
- Hypertension	534	80.7%	39.5%
- Diabetes	534	23.8%	42.6%
- Chronic kidney disease	534	1.5%	11.4
- Epilepsy	534	1.3%	10.6%
- Asthma	534	2.8%	16.5%
- HIV	534	4.3%	20.3%
- TB	534	0.06%	7.5%
- Other	534	7.5%	26.3%
Multiple chronic conditions	534	20.8%	40.6%
Prevented from working			
Completely prevented	534	9.6%	29.4%
Never prevented	534	51.1%	50%
Sometimes prevented	534	39.3%	48.9%
Days missed work (last month)	534	5.15	9.574
Last time sought CD care	504[1]		
- Within the last month		74.8%	43.5%
- Within the last 6 months		13.3%	34%
- More than 6 months ago		11.9%	32.4%
Medicines prescribed during last visit	534	89.1%	31.1%

For peer review only

Medicines taken in the last 7 days	534	68.2%	46.6%
Facility level	.	.	.
Dispensary	1748	9.5%	29.3%
Health centre	1748	67.1%	47%
Hospital	1748	23.4%	42.3%
Closest facility visited	1748	81.4%	38.9%
Usual facility visited	1748	86.7%	34%
Accompanied to the facility	1748	21.9%	41.4%
Transportation mode	.	.	.
Walk	1746	45.1%	49.8%
Bicycle	1746	6.9%	25.4%
Your own motorbike	1746	4.3%	20.3%
Motorbike taxi	1746	29.8%	45.7%
Your own car	1746	2.7%	16.4%
Bus	1746	8.4%	27.8%
Bajaj	1746	2%	14%
A friend or family member brought me	1746	0.3%	5.9%
Other	1746	0.4%	6.3%
Travel time (return) (mins)	1744	98.64	91.31
Clinic time (mins)	1714	130.94	104.17
Travel cost (USD)	1675	1.39	2.81

Footnote, 1: *n* of participants' last time seeking CD care differs from *n* of patients with CDs because for 30 participants, the day of the survey was the day of their first diagnosis

Most patients with CDs appeared adherent to their treatment and follow-up schedule, with 75% seeking care within the last month and 89% received prescription medicines during their last visit whereas 68% took their medicines within the last seven days. Most participants were recruited from a health centre (67.1%) and 21.9% were accompanied to the facility, mostly by one of their children or their partner. Most participants travelled by foot (45%) or motorbike taxi (30%) for more than one and a half hours for the return journey. Including those who travelled by bicycle (6.9%), 52% of patients had no financial costs for travel. In addition to travel time, patients spent more than two hours at the facility, including both waiting and consultation time (Table 2). The average travel time was longer when attending health centres or hospitals compared to dispensaries.

For peer review only

Table 2: Non-medical costs of care-seeking and productivity costs of illness[3], estimated using the human capital method.

	Direct Non-Medical Cost			Indirect Non-Medical Cost			Total Non-Medical Cost			Productivity costs of illness (CD patients)		
	N	*Mean (SD)*	*p-value*	*N*	*Mean (SD)*	*p-value*	*N*	*Mean (SD)*	*p-value*	*N*	*Mean (SD)*	*p-value*
All	1675	1.39 (2.81)		1711	0.87 (0.56)		1638	2.26 (3.02)		534	10.44 (19.41)	
Chronic Condition			***			***			***			
- No	1165	1.22 (2.68)		1189	0.80 (0.50)		1140	2.03 (2.82)			.	
- Yes	510	1.76 (3.05)		522	1.01 (0.66)		498	2.79 (3.36)			.	
Multimorbidity			***			***			***			***
- No	1568	1.35 (2.82)		1603	0.84 (0.53)		1534	2.20 (3.00)		423	9.53 (18.92)	
- Yes	107	1.92 (2.54)		108	1.20 (0.84)		104	3.16 (3.12)		111	13.92 (20.87)	
Insurance status						***			***			**
- Uninsured	1012	1.42 (3.08)		1029	0.78 (0.53)		984	2.22 (3.28)		238	12.94 (21.37)	
- Insured	662	1.34 (2.33)		681	0.99 (0.58)		653	2.33 (2.57)		296	8.43 (17.45)	
Sex												
- Female	1074	1.43 (2.81)		1092	0.87 (0.58)		1052	2.32 (3.04)		363	10.17 (19.18)	
- Male	601	1.31 (2.80)		619	0.85 (0.52)		586	2.17 (2.97)		171	11.01 (19.93)	
Accompanier			***			***			***			**
- No	1320	1.18 (2.62)		1335	0.84 (0.55)		1290	2.03 (2.82)		405	8.80 (17.65)	
- Yes	355	2.15 (3.32)		376	0.95 (0.57)		348	3.12 (3.52)		129	15.59 (23.44)	
Facility Level			***			***			***			
- Dispensary	158	0.64 (1.50)		166	0.52 (0.44)		158	1.15 (1.70)		31	3.79 (10.93)	
- Health Centre	1124	1.46 (3.01)		1150	0.86 (0.52)		1101	2.34 (3.16)		327	11.29 (20.10)	
- Hospital	393	1.47 (2.58)		395	1.02 (0.64)		379	2.50 (2.92)		176	10.03 (19.09)	
Occupation						**						***
- Formal	210	1.11 (2.29)		227	0.81 (0.49)		207	1.92 (2.50)		51	1.99 (5.20)	
- Farmer	906	1.60 (3.24)		913	0.85 (0.58)		884	2.47 (3.44)		314	10.48 (19.05)	
- Self-employed	288	1.20 (2.26)		288	0.87 (0.58)		280	2.10 (2.54)		64	8.20 (17.08)	
- Other	266	1.10 (1.96)		278	0.94 (0.53)		262	2.03 (2.20)		100	14.70 (23.32)	

Note: * p <.05 ** p <.01 *** p <.001

[3] Direct Non-Medical Costs are defined as those costs that are not part of the medical bills from the facility, but are still incurred when accessing care including costs for transportation, food, and accommodation. In our study, only transportation costs are part of this category. Indirect Non-Medical Costs refer to the time losses associated with seeking out medical care, including travel time and time spent at the facility waiting and receiving care. Even though the latter are also productivity losses, we specifically refer here to productivity costs (of illness) as the lost wages that are a result of not being able to work due to (chronic) illness.

For peer review only

Total non-medical costs averaged $2.26 (SD: $3.02), with an average direct cost of $1.39 (SD: $2.81) and indirect cost of $0.87 (SD: 0.56) (Table 2). The sub-analysis of self-reported forgone wages indicated an average opportunity cost of $21.88 (SD: $1180.83) (Table S2). NCD patients incurred significantly higher direct, indirect and total costs relative to other patients (Table 2, Figure 1). Furthermore, patients with comorbid chronic conditions spend more time seeking care compared to those with one condition and in turn bear higher opportunity costs. We also observed that those with insurance spend both more time travelling to the facility and more time at the facility. Sensitivity analyses indicated that with the introduction of a 718 TZS minimum hourly wage, the increased value of time spent travelling and at the clinic leads to a 15.5% increase in the total non-medical cost of seeking care to $2.91 per care-seeking episode (Table S2).

Our analysis using mixed-effects multivariate linear regression revealed that having health insurance, hypertension, or multiple chronic conditions are associated with higher non-medical costs of care (Table 3). Specifically, patients with health insurance pay on average 14% more than uninsured patients, while those with hypertension or multiple chronic conditions pay 14% and 35% more, respectively. Higher education and seeking care at the closest facility to one's residence are associated with 11% lower non-medical costs. However, seeking care at a hospital (as opposed to a dispensary or health centre) is associated with a 67% increase in costs, and being accompanied to the facility is associated with a 39% increase in costs. In addition, we found a significant interaction effect for patients with hypertension who attend hospitals for their care, with costs being on average 35% higher than for other hospital outpatients (Table 4).

Table 3: Mixed-effects linear regression for total non-medical cost, including a random intercept for administrative district.

	Total Non-Medical Cost	95% CI
Health Insurance	0.137**	0.04 – 0.24
	(0.0504)	
Hypertension	0.143*	0.002 – 0.28
	(0.0717)	
Multiple Chronic Conditions	0.354***	0.16 – 0.55
	(0.0998)	
Sex (= male)	0.00384	(0.09) – 0.10
	(0.0487)	
Median Age	-0.0870	(0.19) – 0.02
	(0.0535)	
Higher education	-0.114**	(0.22) – (0.008)
	(0.0543)	
Employed (last 12 months)	0.0353	(0.08) – 0.15
	(0.0561)	

For peer review only - http://bmjopen.bmj.com/site/about/guidelines.xhtml

Closest facility	-0.986*** (0.0584)	(1.10) – (0.87)
Accompanier	0.390*** (0.0561)	0.28 – 0.50
Hospital	0.673*** (0.0915)	0.49 – 0.85
Health Centre	0.567*** (0.0809)	0.41 – 0.73
Hospital#Hypertension	0.352** (0.136)	0.09 – 0.62
Constant	8.115*** (0.0993)	7.92 – 8.31
Observations	1,637	
Clusters (districts)	2	

Standard errors in parentheses
95% CI: negative numbers in parentheses
Note: * p <.05 ** p <.01 *** p <.001

Table 4: Negative binomial regression of the number of days in the past month that NCD patients' illness prevented them from working.

	Number of days missed work	95% CI
Health Insurance	-0.539*** (0.139)	(0.81) – (0.27)
Multiple Chronic Conditions	0.502*** (0.150)	0.21 – 0.80
Medicines prescribed	-0.782** (0.262)	(1.30) – (0.27)
Medicines taken (last 7 days)	0.354 (0.201)	(0.04) – 0.75
Last time sought care		
- Within the last 6 months	-0.423 (0.259)	(0.93) – 0.08
- More than 6 months ago	-0.448 (0.230)	(0.90) – 0.003
Sex (=male)	0.0380 (0.144)	(0.24) – 0.32
Age	0.0284*** (0.00573)	0.02 – 0.04
Higher education	-0.629** (0.207)	(1.03) – (0.22)
Occupation		
- Farmer	0.461 (0.326)	(0.18) – 1.10

For peer review only - http://bmjopen.bmj.com/site/about/guidelines.xhtml

For peer review only

- Self-employed	0.635 (0.363)	(0.08) – 1.35
- Other	0.857* (0.338)	0.19 – 1.52
Constant	0.190 (0.496)	(0.78) – 1.16
Observations	515	

Standard errors in parentheses
95% CI: negative numbers in parentheses
Note: * p <.05 ** p <.01 *** p <.001

We also observed that patients with multiple chronic conditions tend to miss more work days due to illness, while those with insurance or prescription medications tend to miss fewer. More highly educated individuals tend to miss fewer days, while older patients tend to miss more. However, we did not find any significant associations between treatment-seeking behaviour or medication adherence and absenteeism among patients with CDs.

DISCUSSION

The results of this study reveal that patients still incur substantial non-medical costs when seeking health care, equivalent to 114% of the minimum daily wage[4]. These findings are consistent with previous research conducted in Tanzania and in other sub-Saharan African countries. One major contributor to these costs is the time required to reach the healthcare facilities. For instance, another study reported an average of 62 minutes to reach a hospital, compared to nearly 50 minutes in this study.(35) In a sample of 1407 patients requiring maternal and child health care (MCH), the average travel time was 30 minutes for a one-way trip, at an average cost of $0.41.(36) In addition, the reported average time spent at the clinic was almost an hour. These estimates are somewhat lower than our finding, which could be due to the greater and more decentralized availability of MCH services that do not require patients to travel as far. In fact, 70% of those seeking MCH care walked to the facility,(36) which is far higher than those in our sample.

People living with HIV in rural Tanzania cross even larger distances, travelling an average of 2.81 hours in total and staying 2.32 hours at the clinic, but spend slightly less on transportation ($1.09) than in

[4] Considering a minimum hourly wage of $0.22 and nine working hours per day results in a daily wage of $1.98. The total non-medical cost was on average $2.26; yielding a cost to wage ratio of approximately 114%.

For peer review only - http://bmjopen.bmj.com/site/about/guidelines.xhtml

our sample.(11) The opportunity cost of illness was also lower ($3.79), with patients being ill for 16 hours per year on average, which is potentially due to the restriction of the sample to stable HIV patients. Specifically for patients with cardiovascular diseases, another study reported annual transport costs of $14 in rural areas and $24 in urban areas, with average waiting times of 2 hours and 4 hours respectively.(27) In addition, they reported annual income losses of $23 and $30 per year, respectively. However, these figures are difficult to compare to those of this study because we estimated costs per visit rather than annually. In addition, this study only had a rather small sample size of 100 patients, and included only four health facilities. Highly educated individuals incur lower time and travel costs in our sample, similar to other findings.(37) We suspect that this is a result of highly educated people being less likely to be engaged in strenuous physical labour and their proximity to health facilities, given that they tend to live in urban areas with high densities of facilities.

Our study revealed that health insurance status has contrasting effects on direct and indirect non-medical cost, with a non-significant negative association with travel cost and a strongly significant positive association with time cost, both with the travel and clinic time. This could result from the fact that a higher proportion of insured people seek care at the hospital. Since there are only a few hospitals, most people would have to travel further to reach them. . In addition, they are often more crowded, leading to longer waiting times and higher time costs.(27)

The financial burden of seeking healthcare does not solely fall on patients, but also their informal caregivers. Our study shows that having an accompanier to bring patients to healthcare facilities is associated with significantly higher direct and indirect non-medical costs, even without accounting for the caregiver cost. This might be due to the most severely ill being significantly more likely to require accompaniment in order to access the services they need. Those accompanied by an informal caregiver are often unable to walk to the facility and the care they need may not be available at their nearest facility, resulting in higher travel and time costs (Table 2). The lack of previous studies with which to compare our findings demonstrates the novelty of our work. However, a study conducted in Ghana supports the interpretation of our findings in that patients with CDs were shown to use accompaniers in order to overcome mobility and transport barriers to reaching more distant tertiary care facilities,(38) thus explaining why those with accompaniers incur higher direct and indirect non-medical costs than those without. Additionally, the majority of participants reported their co-resident household members, such as their partner or child, as informal caregivers, indicating that these households face a double burden.

Patients with CDs face such costs even more frequently than other patients, due to the need for monthly treatment and the frequent stockouts of essential medicines, which potentially explains why

For peer review only

For peer review only - http://bmjopen.bmj.com/site/about/guidelines.xhtml

most survey participants' last doctor's visit occurred within the last month.(13, 39) Therefore, improving accessibility of NCD care at primary and secondary care levels can reduce non-medical costs and improve the availability of medicines at these levels of care. This would allow patients to receive longer-term prescriptions, requiring fewer visits to the health care facility to refill medications. Decentralizing NCD services would not only provide more affordable care for NCD patients but would result in cost savings for insurers and the health system,(40) while generating additional revenue for primary and secondary care facilities.

Furthermore, it is noteworthy that the opportunity cost associated with illness-related missed work surpasses the non-medical cost for accessing care, and is significantly higher for uninsured patients than insured ones, as they on average miss two more days of work per month. Absenteeism thus affects the uninsured more than the insured, knowing that direct OOP payments introduce a substantial financial barrier to accessing care.(41) However, our results suggest that engagement in care is associated with fewer missed work days. Moreover, retired, and hence older individuals, are most often unable to carry out their daily tasks, while those in formal employment seem to be the least impacted.

Our estimates of absenteeism due to chronic illness are higher than those reported in other studies. For instance, a study in Namibia found an average of only one day of sick leave over a 90-day period in employer records.(42) However, their study included both sick and non-sick employees, while our absenteeism data focused only on patients with chronic illnesses. Additionally, employer records may not capture the missed workdays of informal workers who make up the majority of Tanzania's workforce, particularly in the agriculture sector, where physically strenuous work is more likely to be impacted by chronic illness. To reduce the access and opportunity costs for NCD patients, it would be important to prioritize the provision of basic NCD services at health centres and dispensaries and actively promote patients' engagement in care.

The study findings should be interpreted in the light of some limitations. The key limitation is the lack of reliable self-reported individual income estimates, which required us to rely upon the human capital method. In the supplementary materials, we provided an overview of the costs, as presented in Table 2, including the self-reported lost income due to seeking care on the day of the survey. These figures suggest that our estimates potentially underestimate the indirect non-medical cost and opportunity costs. However, participants are likely to overestimate their lost income, so we suspect that the actual cost lies somewhere in between. Other limitations are that travel, clinic time and number of missed days of work were self-reported and that the use of multiple modes of transportation was not considered when assessing travel costs. Additionally, exit surveys do not capture individuals with NCDs

For peer review only - http://bmjopen.bmj.com/site/about/guidelines.xhtml

who do not seek healthcare at all, potentially hindered by the high cost burden. However, it does minimize recall bias related to the reported time and cost variables. While minimum wages have increased since the data collection for this study in 2020/2021, corresponding acceleration of inflation and fuel price increases may have negated any potential improvements to the affordability of the direct non-medical costs of seeking care.

In addition, we were unable to explore distress-financing strategies such as borrowing money or selling assets, nor could we stratify costs by socioeconomic status. Lastly, research has shown that the direct and indirect costs of seeking care are higher during the rainy season,(43) as our data collection occurred during the dry season, we would expect the travel time to be higher during other times of the year.(44) Given that most patients were subsistence farmers, they potentially not only experience seasonality in their income, but also in their medical costs, which was not captured in this study.

Despite these limitations, this study sheds light on the challenges faced by Tanzanian patients with chronic conditions seeking care at health facilities, especially in rural districts. The study suggests that decentralizing the provision of NCD care from hospitals to health centres and dispensaries may be beneficial. This approach could help to reduce patients' non-medical and opportunity costs associated with travel and increase their engagement in care.

For peer review only

For peer review only - http://bmjopen.bmj.com/site/about/guidelines.xhtml

REFERENCES

1. United Nations. Transforming our world: the 2030 Agenda for Sustainable Development. 2015. Available from: https://www.un.org/ga/search/view_doc.asp?symbol=A/RES/70/1&Lang=E.

2. Jan S, Laba TL, Essue BM, Gheorghe A, Muhunthan J, Engelgau M, et al. Action to address the household economic burden of non-communicable diseases. Lancet. 2018;391(10134):2047-58. doi:10.1016/S0140-6736(18)30323-4.

3. Jakovljevic M, Jakab M, Gerdtham U, McDaid D, Ogura S, Varavikova E, et al. Comparative financing analysis and political economy of noncommunicable diseases. Journal of Medical Economics. 2019;22(8):722-7. doi:10.1080/13696998.2019.1600523.

4. Adinan J, Manongi R, Temu GA, Kapologwe N, Marandu A, Wajanga B, et al. Preparedness of health facilities in managing hypertension & diabetes mellitus in Kilimanjaro, Tanzania: a cross sectional study. BMC Health Services Research. 2019;19(1). doi:10.1186/s12913-019-4316-6.

5. Siddharthan T, Ramaiya K, Yonga G, Mutungi GN, Rabin TL, List JM, et al. Noncommunicable Diseases In East Africa: Assessing The Gaps In Care And Identifying Opportunities For Improvement. Health Affairs. 2015;34(9):1506-13. doi:10.1377/hlthaff.2015.0382.

6. Bermudez-Tamayo C, Besançon S, Johri M, Assa S, Brown JB, Ramaiya K. Direct and indirect costs of diabetes mellitus in Mali: A case-control study. PLOS ONE. 2017;12(5):e0176128. doi:10.1371/journal.pone.0176128.

7. Mutyambizi C, Pavlova M, Chola L, Hongoro C, Groot W. Cost of diabetes mellitus in Africa: a systematic review of existing literature. Globalization and Health. 2018;14(1):3. doi:10.1186/s12992-017-0318-5.

8. Chimbindi N, Bor J, Newell M-L, Tanser F, Baltussen R, Hontelez J, et al. Time and Money: The True Costs of Health Care Utilization for Patients Receiving "Free" HIV/Tuberculosis Care and Treatment in Rural KwaZulu-Natal. JAIDS Journal of Acquired Immune Deficiency Syndromes. 2015;70(2):e52. doi:10.1097/QAI.0000000000000728.

9. Mhalu G, Hella J, Mhimbira F, Said K, Mosabi T, Mlacha YP, et al. Pathways and associated costs of care in patients with confirmed and presumptive tuberculosis in Tanzania: A cross-sectional study. BMJ Open. 2019;9(4):e025079. doi:10.1136/bmjopen-2018-025079.

10. Mudzengi D, Sweeney S, Hippner P, Kufa T, Fielding K, Grant AD, et al. The patient costs of care for those with TB and HIV: a cross-sectional study from South Africa. Health Policy Plan. 2017;32(suppl_4):iv48-iv56. doi:10.1093/heapol/czw183.

11. Okere NE, Corball L, Kereto D, Hermans S, Naniche D, Rinke de Wit TF, et al. Patient-incurred costs in a differentiated service delivery club intervention compared to standard clinical care in Northwest Tanzania. J Int AIDS Soc. 2021;24(6):e25760. doi:10.1002/jia2.25760.

12. Bommer C, Heesemann E, Sagalova V, Manne-Goehler J, Atun R, Bärnighausen T, et al. The global economic burden of diabetes in adults aged 20–79 years: a cost-of-illness study. The Lancet Diabetes & Endocrinology. 2017;5(6):423-30. doi:10.1016/S2213-8587(17)30097-9.

13. Macha J, Harris B, Garshong B, Ataguba JE, Akazili J, Kuwawenaruwa A, et al. Factors influencing the burden of health care financing and the distribution of health care benefits in

For peer review only

For peer review only - http://bmjopen.bmj.com/site/about/guidelines.xhtml

Ghana, Tanzania and South Africa. Health Policy and Planning. 2012;27(suppl_1):i46-i54. doi:10.1093/heapol/czs024.

14. Varela C, Young S, Mkandawire N, Groen RS, Banza L, Viste A. TRANSPORTATION BARRIERS TO ACCESS HEALTH CARE FOR SURGICAL CONDITIONS IN MALAWI a cross sectional nationwide household survey. BMC Public Health. 2019;19(1):264. doi:10.1186/s12889-019-6577-8.

15. Kirigia JM, Sambo HB, Sambo LG, Barry SP. Economic burden of diabetes mellitus in the WHO African region. BMC Int Health Hum Rights. 2009;9:6. doi:10.1186/1472-698X-9-6.

16. Wang Q, Brenner S, Kalmus O, Banda HT, De Allegri M. The economic burden of chronic non-communicable diseases in rural Malawi: an observational study. BMC Health Services Research. 2016;16(1):457. doi:10.1186/s12913-016-1716-8.

17. Carls GS, Roebuck MC, Brennan TA, Slezak JA, Matlin OS, Gibson TB. Impact of medication adherence on absenteeism and short-term disability for five chronic diseases. Journal of Occupational and Environmental Medicine. 2012;54(7):792-805. doi:10.1097/JOM.0b013e31825463e9.

18. Povia JP, Masenga SK, Hamooya BM, Gebremeskel Y. Productivity-adjusted life-years and correlates of uncontrolled hypertension at two health facilities in Zambia. PLoS One. 2023;18(12):e0295401. doi:10.1371/journal.pone.0295401.

19. Jo C. Cost-of-illness studies: concepts, scopes, and methods. Clinical and Molecular Hepatology. 2014;20(4):327. doi:10.3350/cmh.2014.20.4.327.

20. Shephard C, Hirst A, Buckland A, Vlachaki I, Wang-Silvanto J. Three Methods for Measuring Productivity Losses Due to Health Related Presenteeism in Economic Evaluations. Value in Health. 2016;19(7):A364-A5. doi:10.1016/j.jval.2016.09.107.

21. World Health Organization. WHO guide to identifying the economic consequences of disease and injury: World Health Organization; 2009. Available from: https://apps.who.int/iris/handle/10665/137037.

22. Rajabi M, Rezaee M, Omranikhoo H, Khosravi A, Keshmiri S, Ghaedi H, et al. Cost of Illness of COVID-19 and Its Consequences on Health and Economic System. Inquiry. 2022;59:469580221144398. doi:10.1177/00469580221144398.

23. Mogasale V, Kar SK, Kim JH, Mogasale VV, Kerketta AS, Patnaik B, et al. An Estimation of Private Household Costs to Receive Free Oral Cholera Vaccine in Odisha, India. PLoS Negl Trop Dis. 2015;9(9):e0004072. doi:10.1371/journal.pntd.0004072.

24. Gupta P, Singh K, Shivashankar R, Singh K, Vamadevan A, Mohan V, et al. Healthcare utilisation and expenditure patterns for cardio-metabolic diseases in South Asian cities: the CARRS Study. BMJ Open. 2020;10(9):e036317. doi:10.1136/bmjopen-2019-036317.

25. Seuring T, Archangelidi O, Suhrcke M. The Economic Costs of Type 2 Diabetes: A Global Systematic Review. PharmacoEconomics. 2015;33(8):811-31. doi:10.1007/s40273-015-0268-9.

26. World Health Organization Global TB Programme. Protocol for survey to determine direct and indirect costs due to TB and to estimate proportion of TB-affected households experiencing catastrophic total costs due to TB: Field testing version: World Health Organization; 2015. Available from: https://cdn.who.int/media/docs/default-source/hq-tuberculosis/protocol-for-survey-to-determine-direct.pdf?sfvrsn=c1fdb231_5&download=true.

27. Ngalesoni F, Ruhago G, Norheim OF, Robberstad B. Economic cost of primary prevention of cardiovascular diseases in Tanzania. Health Policy Plan. 2015;30(7):875-84. doi:10.1093/heapol/czu088.

For peer review only - http://bmjopen.bmj.com/site/about/guidelines.xhtml

28. Osetinsky B, Mhalu G, Mtenga S, Tediosi F. Care cascades for hypertension and diabetes: Cross-sectional evaluation of rural districts in Tanzania. PLOS Medicine. 2022;19(12):e1004140. doi:10.1371/journal.pmed.1004140.
29. Geldsetzer P, Fink G, Vaikath M, Bärnighausen T. Sampling for Patient Exit Interviews: Assessment of Methods Using Mathematical Derivation and Computer Simulations. Health Services Research. 2018;53(1):256-72. doi:10.1111/1475-6773.12611.
30. Government of the United Republic of Tanzania. Government Notice No. 196: Labour Institutions Wage Order. Gazette of the United Republic of Tanzania. 2013;26(94): Supplement No. 24. Available from: https://www.kazi.go.tz/uploads/documents/en-1599781960-WAGE%20ORDER,196.pdf.
31. Bank of Tanzania. Previous Exchange Rates [Available from: https://www.bot.go.tz/ExchangeRate/previous_rates.
32. Government of the United Republic of Tanzania. Government Notice No. 687: Labour Institutions Wage Order. Gazette of the United Republic of Tanzania. 2022;45(103): Supplement No. 45
Available from: https://oagmis.agctz.go.tz/portal/legislation/1174/download.
33. StataCorp. Stata Stastical Software: Release 16. College Station, TX: StataCorp LLC; 2019. Available from https://www.stata.com/.
34. R Core Team. R: A language and environment for statistical computing. Vienna, Austria: R Foundation for Statistical Computing; 2022. Available from: https://www.R-project.org/.
35. Wong KLM, Brady OJ, Campbell OMR, Banke-Thomas A, Benova L. Too poor or too far? Partitioning the variability of hospital-based childbirth by poverty and travel time in Kenya, Malawi, Nigeria and Tanzania. International Journal for Equity in Health. 2020;19(1):15. doi:10.1186/s12939-020-1123-y.
36. Binyaruka P, Borghi J. An equity analysis on the household costs of accessing and utilising maternal and child health care services in Tanzania. Health Economics Review. 2022;12(1). doi:10.1186/s13561-022-00387-7.
37. Butt MD, Ong SC, Wahab MU, Rasool MF, Saleem F, Hashmi A, et al. Cost of Illness Analysis of Type 2 Diabetes Mellitus: The Findings from a Lower-Middle Income Country. International Journal of Environmental Research and Public Health. 2022;19(19):12611. doi:10.3390/ijerph191912611.
38. Dowou RK, Amu H, Saah FI, Arthur LE, Dotse PAN, Bain LE. Management of chronic non-communicable diseases in Ghana: a qualitative study of patients' coping strategies and the role of caregivers. BMC Health Services Research. 2023;23(1):371. doi:10.1186/s12913-023-09398-4.
39. Kuwawenaruwa A, Wyss K, Wiedenmayer K, Metta E, Tediosi F. The effects of medicines availability and stock-outs on household's utilization of healthcare services in Dodoma region, Tanzania. Health Policy Plan. 2020;35(3):323-33. doi:10.1093/heapol/czz173.
40. Hooley B, Osetinsky B, Tediosi F, editors. The cost of overutilizing hospitals for diabetes and hypertension care in Tanzania. iHEA; 2021 2021.
41. Huffman MD, Rao KD, Pichon-Riviere A, Zhao D, Harikrishnan S, Ramaiya K, et al. A Cross-Sectional Study of the Microeconomic Impact of Cardiovascular Disease Hospitalization in Four Low- and Middle-Income Countries. PLoS One. 2011;6(6):e20821. doi:10.1371/journal.pone.0020821.

For peer review only - http://bmjopen.bmj.com/site/about/guidelines.xhtml

42. Guariguata L, de Beer I, Hough R, Bindels E, Weimers-Maasdorp D, Feeley FG, et al. Diabetes, HIV and other health determinants associated with absenteeism among formal sector workers in Namibia. BMC Public Health. 2012;12(1):44. doi:10.1186/1471-2458-12-44.

43. Su TT, Pokhrel S, Gbangou A, Flessa S. Determinants of household health expenditure on western institutional health care. Eur J Health Econ. 2006;7(3):195-203. doi:10.1007/s10198-006-0354-1.

44. Makanga PT, Schuurman N, Sacoor C, Boene HE, Vilanculo F, Vidler M, et al. Seasonal variation in geographical access to maternal health services in regions of southern Mozambique. International Journal of Health Geographics. 2017;16(1). doi:10.1186/s12942-016-0074-4.

FIGURE LEGEND

Figure 1: Panel A, Total non-medical costs by health facility level and NCD status. Panel B, Total non-medical costs by health facility level and NCD status, including caregiver cost.

AUTHORS' CONTRIBUTION

AV, BH and FT developed the research design. AV and BH drafted the initial version of the manuscript and performed the analysis under the supervision of FT. KT and GM provided inputs on the methodology and implemented the data collection. All authors contributed to the article and approved the submitted version.

ACKNOWLEDGEMENTS

The authors wish to acknowledge the field workers of the Ifakara Health Institute for their data collection efforts, and the stakeholders and policymakers who attended dissemination workshops in Dodoma and Dar es Salaam whose discussions provided valuable input and context to this work.

COMPETING INTERESTS

The authors have no competing interests to declare.

FUNDING STATEMENT

This work was funded by the Swiss Programme for Research on Global Issues for Development (Grant number: 183760), a joint funding initiative by the Swiss Agency for Development and Cooperation (SDC) and the Swiss National Science Foundation (SNSF). The funders had no role in study design, data collection and analysis, decision to publish, or preparation of the manuscript.

For peer review only

DATA AVAILABILITY STATEMENT

Data are available upon reasonable request. According to the institutional review board of IHI, we are not allowed to make the data publicly available. Interested researchers should contact the corresponding author.

For peer review only - http://bmjopen.bmj.com/site/about/guidelines.xhtml

For peer review only

For peer review only - http://bmjopen.bmj.com/site/about/guidelines.xhtml

For peer review only

For peer review only - http://bmjopen.bmj.com/site/about/guidelines.xhtml

For peer review only

For peer review only - http://bmjopen.bmj.com/site/about/guidelines.xhtml

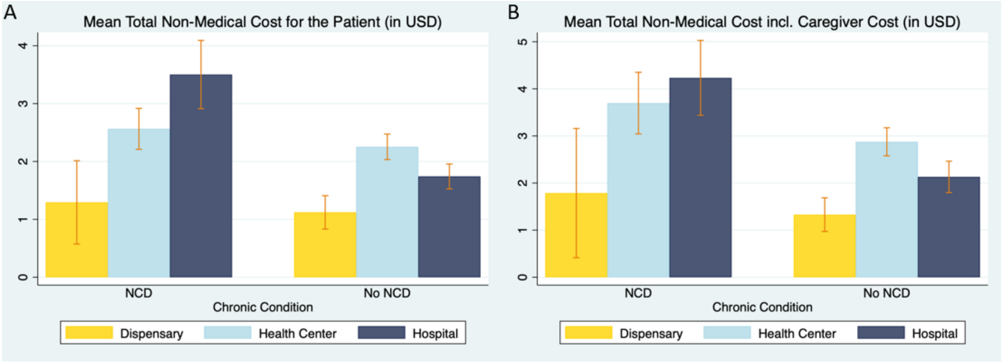

738x267mm (38 x 38 DPI)

SUPPLEMENTARY MATERIALS

Supplementary tables

Table S1: Descriptive statistics, disaggregated according to whether patients were accompanied by a caregiver.

		Accompanier		No Accompanier		
Variable	Obs.	Mean	Std. Dev.	Mean	Std. Dev.	p-value
Sex (male)	383	**.264**	.441	**.39**	.488	**<0.001**
Education level	.	.	.	.	.	0.083
Never attended school	383	.073	.261	.042	.202	
Some primary school	383	.123	.329	.122	.327	
**Completed primary school**	383	**.475**	.5	**.545**	.498	
Some secondary school	383	.047	.212	.035	.184	
Completed secondary	383	.219	.414	.188	.391	
Completed college education	383	.047	.212	.049	.216	
Completed university education	383	.016	.124	.018	.134	
Working (last 12 months)	383	.159	.366	.298	.458	**<0.001**
Occupation	.	.	.	.	.	**<0.001**
Public servant	383	.039	.194	.056	.229	
Private formal sector	383	.018	.134	.097	.296	
**Subsistence farmer**	383	**.527**	.5	**.532**	.499	
Large-scale farming	383	.005	.072	.004	.06	
Self-employed/small business	383	.146	.354	.171	.377	
Self-employed/large business	383	.003	.051	.004	.06	
Taking care of home and/or children	383	.102	.303	.033	.179	
Student	383	.055	.228	.036	.186	
Retired	383	.065	.247	.041	.198	
Other	383	.039	.194	.027	.162	
Marital status	.	.	.	.	.	**0.004**
**Married**	383	**.627**	.484	**.655**	.476	
Living with partner	383	.042	.2	.021	.144	
Divorced	383	.008	.088	.007	.085	
Separated	383	.021	.143	.054	.227	
Widowed	383	.151	.359	.107	.309	
Never married	383	.151	.359	.155	.362	
Age	383	43.919	19.048	44.195	16.202	0.78
Health insurance	383	.368	.483	.402	.491	0.22
Any SHP	383	.428	.495	.484	.5	0.055
Any chronic condition	383	.337	.473	.297	.457	0.13
Type of chronic condition						
- Hypertension	129	.861	.348	.790	.408	0.078
- Diabetes	129	.217	.414	.244	.430	0.52
- Chronic kidney disease	129	.031	.174	.007	.086	0.040
- Epilepsy						
- Asthma	129	.0	.0	.015	0.121	0.16
- HIV	129	.039	.194	.025	.155	0.40
- TB	129	.031	.174	.047	.212	0.44
- Other	129	.0	.0	.007	.086	0.33

For peer review only

	129	.093	.292	.069	.254	0.37
Multiple chronic conditions	129	.24	.429	.198	.399	0.30
Prevented from working						**<0.001**
Completely prevented	129	.442	.499	.533	.5	
Never prevented	129	.186	.391	.067	.25	
Sometimes prevented	129	.372	.485	.4	.491	
Days missed work	129	7.69	11.565	4.341	8.708	**<0.001**
Facility level	.	.	.	.	.	**0.003**
Dispensary	383	.081	.273	.099	.299	
**Health centre**	383	**.742**	.438	**.651**	.477	
Hospital	383	.178	.383	.25	.433	
Closest facility visited	383	.791	.407	.888	.316	**<0.001**
Normal facility visited	383	.749	.434	.832	.374	**<0.001**
Transportation mode	.	.	.	.	.	**<0.001**
**Walk**	383	**.295**	.457	**.494**	.5	
Bicycle	383	.063	.243	.071	.257	
Your own motorbike	383	.078	.269	.033	.179	
Motorbike taxi	383	.36	.481	.28	.449	
Your own car	383	.06	.238	.018	.134	
Bus	383	.084	.277	.084	.278	
Bajaj	383	.037	.188	.015	.123	
A friend or family member brought me	383	.01	.102	.001	.038	
Other	383	.013	.114	.001	.038	
Travel time (return) (mins)	381	117.85	102.23	93.28	87.30	**<0.001**
Clinic time (mins)	378	134.45	103.06	129.94	104.50	0.46

For peer review only

Table S2 Non-medical costs of care-seeking and opportunity costs of illness, estimated using both the human capital method and self-reported lost earnings

	Direct Non-Medical Cost			Indirect Non-Medical Cost			Total Non-Medical Cost			Opportunity costs of illness (CD patients)		
	N	Mean (SD)	p-value	N	Mean (SD)	p-value	N	Mean (SD)	p-value	N	Mean (SD)	p-value
All	1675	1.39 (2.81)		1617	1.51 (2.13)		1550	2.93 (3.67)		498	21.88 (180.83)	
Chronic Condition			***			*			**			
No	1165	1.22 (2.68)		1117	1.48 (2.04)		1072	2.76 (3.50)			.	
Yes	510	1.76 (3.05)		500	1.58 (2.30)		478	3.32 (3.99)			.	
Multimorbidity			***						**			**
No	1568	1.35 (2.82)		1518	1.50 (2.14)		1454	2.89 (3.66)		399	22.70 (201.31)	
Yes	107	1.92 (2.54)		99	1.67 (1.91)		96	3.60 (3.77)		99	18.56 (35.14)	
Insurance status						***			***			*
Uninsured	1012	1.42 (3.08)		960	1.32 (1.72)		920	2.80 (3.72)		219	14.56 (24.80)	
Insured	662	1.34 (2.33)		656	1.79 (2.59)		629	3.12 (3.59)		279	27.62 (240.62)	
Sex						***						
Female	1074	1.43 (2.81)		1055	1.32 (1.80)		1017	2.76 (3.47)		341	11.87 (23.61)	
Male	601	1.31 (2.80)		562	1.86 (2.60)		533	3.26 (4.01)		157	43.61 (319.79)	
Accompanier			***						***			*
No	1320	1.18 (2.62)		1251	1.57 (2.29)		1211	2.79 (3.64)		376	23.87 (207.70)	
Yes	355	2.15 (3.32)		366	1.28 (1.40)		339	3.44 (3.72)		122	15.74 (23.61)	
Facility Level			***			***			***			
Dispensary	158	0.64 (1.50)		159	1.22 (2.29)		151	1.91 (2.99)		31	6.15 (17.39)	
Health Centre	1124	1.46 (3.01)		1122	1.45 (2.00)		1075	2.92 (3.66)		314	13.77 (26.53)	
Hospital	393	1.47 (2.58)		336	1.84 (2.40)		324	3.44 (3.89)		153	41.70 (323.77)	
Occupation						***			***			**
Formal	210	1.11 (2.29)		177	2.96 (3.38)		161	4.15 (4.22)		39	122.78 (639.09)	
Farmer	906	1.60 (3.24)		882	1.17 (1.24)		855	2.81 (3.59)		299	12.20 (25.25)	
Self-employed	288	1.20 (2.26)		280	1.83 (2.88)		272	3.09 (3.92)		58	10.92 (22.66)	
Other	266	1.10 (1.96)		273	1.33 (2.03)		257	2.44 (3.10)		97	16.87 (27.29)	

For peer review only

Table S3: Sensitivity analysis of Table 2 with new minimum hourly wage introduced from January 2023 (718 TZS ~ 0-30 USD)

	**Direct Non-Medical Cost**			**Indirect Non-Medical Cost**			**Total Non-Medical Cost**			**Estimated Lost Income (NCD patients)**		
	N	Mean (SD)	p-value	N	Mean (SD)	p-value	N	Mean (SD)	p-value	N	Mean (SD)	p-value
All	1675	1.39 (2.81)		1711	1.21 (0.78)		1638	2.61 (3.11)		534	14.62 (27.17)	
Chronic Condition			***			***			***			
No	1165	1.22 (2.68)		1189	1.12 (0.70)		1140	2.35 (2.89)			.	
Yes	510	1.76 (3.05)		522	1.41 (0.92)		498	3.20 (3.51)			.	
Multimorbidity			***			***			***			***
No	1568	1.35 (2.82)		1603	1.18 (0.74)		1534	2.54 (3.08)		423	13.34 (26.49)	
Yes	107	1.92 (2.54)		108	1.68 (1.17)		104	3.65 (3.37)		111	19.48 (29.22)	
Insurance status						***			***			**
Uninsured	1012	1.42 (3.08)		1029	1.10 (0.74)		984	2.53 (3.37)		238	18.11 (29.91)	
Insured	662	1.34 (2.33)		681	1.39 (0.82)		653	2.73 (2.69)		296	11.80 (24.44)	
Sex												
Female	1074	1.43 (2.81)		1092	1.22 (0.81)		1052	2.67 (3.15)		363	14.24 (26.85)	
Male	601	1.31 (2.80)		619	1.19 (0.73)		586	2.51 (3.05)		171	15.42 (27.90)	
Accompanier			***			***			***			**
No	1320	1.18 (2.62)		1335	1.18 (0.78)		1290	2.37 (2.92)		405	12.32 (24.71)	
Yes	355	2.15 (3.32)		376	1.33 (0.80)		348	3.50 (3.62)		129	21.82 (32.82)	
Facility Level			***			***			***			
Dispensary	158	0.64 (1.50)		166	0.73 (0.61)		158	1.36 (1.81)		31	5.31 (15.30)	
Health Centre	1124	1.46 (3.01)		1150	1.20 (0.73)		1101	2.68 (3.23)		327	15.80 (28.14)	
Hospital	393	1.47 (2.58)		395	1.43 (0.90)		379	2.92 (3.07)		176	14.05 (26.73)	
Occupation						**						***
Formal	210	1.11 (2.29)		227	1.14 (0.69)		207	2.25 (2.61)		51	2.78 (7.28)	
Farmer	906	1.60 (3.24)		913	1.20 (0.81)		884	2.81 (3.53)		314	14.67 (26.68)	
Self-employed	288	1.20 (2.26)		288	1.22 (0.81)		280	2.45 (2.67)		64	11.49 (23.91)	
Other	266	1.10 (1.96)		278	1.31 (0.74)		262	2.40 (2.32)		100	20.58 (32.65)	

For peer review only

Table S4: Sensitivity analysis of mixed-effects linear regression for total non-medical cost, including a random intercept for administrative district

	Total Non-Medical Cost	95% CI
Health Insurance	0.136**	0.05 – 0.23
	(0.046)	
Hypertension	0.133*	0.005 – 0.26
	(0.065)	
Multiple Chronic Conditions	0.32***	0.14 – 0.50
	(0.091)	
Sex (= male)	0.01	(0.08) – 0.10
	(0.044)	
Median Age	-0.077	(0.17) – 0.02
	(0.049)	
Higher education	-0.115**	(0.21) – (0.018)
	(0.049)	
Employed (last 12 months)	0.027	(0.07) – 0.13
	(0.0561)	
Closest facility	-0.898***	(1.00) – (0.79)
	(0.0584)	
Accompanier	0.390***	0.25 – 0.45
	(0.051)	
Hospital	0.649***	0.49 – 0.81
	(0.083)	
Health Center	0.546***	0.40 – 0.69
	(0.074)	
Hospital#Hypertension	0.321**	0.08 – 0.56
	(0.123)	
Constant	8.312***	8.13 – 8.49

For peer review only

	(0.09)
Observations	1,637
Clusters (districts)	2

Standard errors in parentheses
95% CI: negative numbers in parentheses
Note: * p <.05 ** p <.01 *** p <.001

For peer review only

Author reflexivity statement

1. How does this study address local research and policy priorities?

This study directly addresses the as-yet underappreciated issue of indirect costs of accessing healthcare in Tanzania, which disproportionately affects Tanzania's majority rural population. This study brings attention to this local issue, and makes recommendations for improving the equity of access to NCD care in Tanzania.

2. How were local researchers involved in study design?

Local researchers KT and GM were involved in the design of the greater research project within which this study is nested, in addition to having been involved in the design of the methodology for the present study. They both have rich experience in international research partnerships, completing and having completed their doctorate at the University of Basel, respectively. They represent a gender- and seniority-balanced local research team composition.

3. How has funding been used to support the local research team?

This study was nested within the broader "Health systems governance for an inclusive and sustainable social health protection in Ghana and Tanzania" study, funded by the Swiss programmes for research on global issues for development that was jointly obtained by Switzerland- and Tanzania-based co-PIs, Prof. Dr. Fabrizio Tediosi and Dr. Sally Mtenga respectively. Throughout the 6-year lifespan of this project, this grant supported equal numbers of Switzerland- and Tanzania-based doctoral students and researchers.

4. How are research staff who conducted data collection acknowledged?

This study recruited research assistants to perform data collection, who are appropriate credited in the acknowledgements section.

5. Do all members of the research partnership have access to study data?

All members of the partnership have access to the study data.

6. How was data used to develop analytical skills within the partnership?

Both Switzerland- and Tanzania-based collaborated on the design of data collection tools, further developing their skills in programming ODK forms. Both Tanzania- and Switzerland-based co-authors had the opportunity to follow courses at the University of Basel to develop the analytical and data analysis skills, while directly working with the data stemming from this study.

7. How have research partners collaborated in interpreting study data?

Authors BH and KT worked together on the cleaning and initial interpretation of study data.

8. How were research partners supported to develop writing skills?

For peer review only

The first authors of this manuscript are junior academics, who received support from senior academic FT throughout the writing process.

9. How will research products be shared to address local needs?

This work has been directly disseminate via two stakeholder panel discussions and presentations with stakeholders and policymakers from the Ministry of Health, Community Development, Elderly and Children, the President's Office for Regional Administration and Local Government, and the Tanzanian National Health Insurance Fund. Both events were attended by local journalists and media, which helped ensure a wider local dissemination of findings.

10. How is the leadership, contribution and ownership of this work by LMIC researchers recognised within the authorship?

Authors RM and NO worked as part of the senior authorship team in developing this manuscript, and their contribution has been recognised as joint first and joint last authors respectively. We have specifically included researchers based in the global south (RM and NO) within the senior authorship team as joint first and joint last authors. We acknowledge, however, that the authorship team is predominantly based in high-income countries. The primary reason for this is that the initiative has been driven from the perspective of journal editors with insight on how to develop transparent and implementable guidelines to assess manuscript submissions from international partnerships.

11. **How have early career researchers across the partnership been included within the authorship team?**

We have included early career researchers (CK, SS & MS) within the authorship team. They attended all the workshops, contributed to the literature review and evidence synthesis and to development of the consensus recommendations. We acknowledge that they are based in high-income countries.

12. How has gender balance been addressed within the authorship?

Three co-authors are men (BH, KT and FT) and two co-authors women (AV, GM).

13. How has the project contributed to training of LMIC researchers?

All researchers based in Tanzania (KT and GM) received or are receiving doctoral training and education in Switzerland, at the University of Basel, financed by the Research for development grant described in point number three.

14. How has the project contributed to improvements in local infrastructure?

This project did not directly contribute to improving local infrastructure.

15. What safeguarding procedures were used to protect local study participants and researchers?

For peer review only

All participants provided informed consent prior to participation. Research protocols were critically reviewed and approved by the institutional review board of the Ifakara Health Institute and the National Institute for Medical Research of Tanzania.

For peer review only

For peer review only

For peer review only

STROBE 2007 (v4) Statement—Checklist of items that should be included in reports of *cross-sectional studies*

Section/Topic	Item #	Recommendation	Reported on page #
**Title and abstract**	1	(*a*) Indicate the study's design with a commonly used term in the title or the abstract	Pages 1 and 2
		(*b*) Provide in the abstract an informative and balanced summary of what was done and what was found	Page 2
**Introduction**			
Background/rationale	2	Explain the scientific background and rationale for the investigation being reported	Pages 4 and 5
Objectives	3	State specific objectives, including any prespecified hypotheses	Page 5
**Methods**			
Study design	4	Present key elements of study design early in the paper	Page 6
Setting	5	Describe the setting, locations, and relevant dates, including periods of recruitment, exposure, follow-up, and data collection	Pages 6 and 7
Participants	6	(*a*) Give the eligibility criteria, and the sources and methods of selection of participants	Page 6
Variables	7	Clearly define all outcomes, exposures, predictors, potential confounders, and effect modifiers. Give diagnostic criteria, if applicable	Pages 6 and 7
Data sources/ measurement	8*	For each variable of interest, give sources of data and details of methods of assessment (measurement). Describe comparability of assessment methods if there is more than one group	Page 6, see also: Osetinsky B, Mhalu G, Mtenga S, Tediosi F. 2022. Care cascades for hypertension and diabetes: Cross-sectional evaluation of rural districts in Tanzania. *PLOS Medicine*, **19**: e1004140.
Bias	9	Describe any efforts to address potential sources of bias	Pages 5, 6 and 7
Study size	10	Explain how the study size was arrived at	Page 6, see also: Osetinsky B, Mhalu G, Mtenga S, Tediosi F. 2022. Care cascades

For peer review only

			for hypertension and diabetes: Cross-sectional evaluation of rural districts in Tanzania. *PLOS Medicine*, **19**: e1004140.
Quantitative variables	11	Explain how quantitative variables were handled in the analyses. If applicable, describe which groupings were chosen and why	Page 7
Statistical methods	12	(*a*) Describe all statistical methods, including those used to control for confounding	Page 7
		(*b*) Describe any methods used to examine subgroups and interactions	Page 7
		(*c*) Explain how missing data were addressed	NA
		(*d*) If applicable, describe analytical methods taking account of sampling strategy	NA
		(*e*) Describe any sensitivity analyses	Page 7, also see supplementary materials
**Results**			
Participants	13*	(a) Report numbers of individuals at each stage of study—eg numbers potentially eligible, examined for eligibility, confirmed eligible, included in the study, completing follow-up, and analysed	Pages 6 and 8
		(b) Give reasons for non-participation at each stage	NA
		(c) Consider use of a flow diagram	NA
Descriptive data	14*	(a) Give characteristics of study participants (eg demographic, clinical, social) and information on exposures and potential confounders	Page 8-9
		(b) Indicate number of participants with missing data for each variable of interest	Pages 9 and 10
Outcome data	15*	Report numbers of outcome events or summary measures	Pages 10-14
Main results	16	(*a*) Give unadjusted estimates and, if applicable, confounder-adjusted estimates and their precision (eg, 95% confidence interval). Make clear which confounders were adjusted for and why they were included	Pages 10-14
		(*b*) Report category boundaries when continuous variables were categorized	NA
		(*c*) If relevant, consider translating estimates of relative risk into absolute risk for a meaningful time period	NA
Other analyses	17	Report other analyses done—eg analyses of subgroups and interactions, and sensitivity analyses	Page 7, also see supplementary materials

For peer review only

**Discussion**			
Key results	18	Summarise key results with reference to study objectives	Page 14
Limitations	19	Discuss limitations of the study, taking into account sources of potential bias or imprecision. Discuss both direction and magnitude of any potential bias	Page 16-17
Interpretation	20	Give a cautious overall interpretation of results considering objectives, limitations, multiplicity of analyses, results from similar studies, and other relevant evidence	Pages 14 and 15
Generalisability	21	Discuss the generalisability (external validity) of the study results	Page 17
**Other information**			
Funding	22	Give the source of funding and the role of the funders for the present study and, if applicable, for the original study on which the present article is based	Page 21

*Give information separately for cases and controls in case-control studies and, if applicable, for exposed and unexposed groups in cohort and cross-sectional studies.

Note: An Explanation and Elaboration article discusses each checklist item and gives methodological background and published examples of transparent reporting. The STROBE checklist is best used in conjunction with this article (freely available on the Web sites of PLoS Medicine at http://www.plosmedicine.org/, Annals of Internal Medicine at http://www.annals.org/, and Epidemiology at http://www.epidem.com/). Information on the STROBE Initiative is available at www.strobe-statement.org.

For peer review only
